# *Lactobacillus*-dominance and rapid stabilization of vaginal microbiota in combined oral contraceptive pill users examined through a longitudinal cohort study with frequent vaginal sampling over two years

**DOI:** 10.1016/j.ebiom.2022.104407

**Published:** 2022-12-16

**Authors:** Susan Tuddenham, Pawel Gajer, Anne E. Burke, Catherine Murphy, Sabra L. Klein, Christina A. Stennett, Barbara Wilgus, Jacques Ravel, Khalil G. Ghanem, Rebecca M. Brotman

**Affiliations:** a Department of Medicine, Johns Hopkins University School of Medicine, Baltimore, MD, USA; b Institute for Genome Sciences, University of Maryland School of Medicine, Baltimore, MD, USA; c Department of Gynecology and Obstetrics, Johns Hopkins University School of Medicine, Baltimore, MD, USA; d W. Harry Feinstone Department of Molecular Microbiology and Immunology, Johns Hopkins Bloomberg School of Public Health, Baltimore, MD, USA; e Department of Epidemiology and Public Health, University of Maryland School of Medicine, Baltimore, MD, USA; f Department of Microbiology and Immunology, University of Maryland School of Medicine, Baltimore, MD, USA

**Keywords:** Hormonal contraception, Vaginal microbiome, Reproductive health, Stability, Obstetrics and gynecology

## Abstract

**Background:**

Bacterial vaginosis (BV), a condition in which vaginal *Lactobacillus* spp. are in low abundance, is associated with vulvovaginal symptoms, obstetric outcomes and urogenital infections. Recurrent BV is difficult to manage, and emerging data indicate a reduced risk of BV with the use of hormonal contraception (HC). Despite widespread use, little longitudinal data is available on whether, and in what timeframe, combined oral contraceptive pills (COCs) may act to affect vaginal microbiota stability and *Lactobacillus* dominance.

**Methods:**

We compared the vaginal microbiota of reproductive-age cisgender women during intervals on combined estrogen and progestin COCs with non-use intervals in a 2-year observational study. Vaginal microbiota were characterized by 16S rRNA gene amplicon sequencing.

**Findings:**

COC users were more likely to have *Lactobacillus*-dominated microbiota and more stable microbiota over time. Stability increased and then plateaued four weeks after COC initiation. The associations between COCs and *Lactobacillus* spp. dominance, and microbiota stability, were statistically significant for White, but not African American women; however sample size was limited for African American participants. Findings were similar for other forms of HC and when excluding samples collected during menses.

**Interpretation:**

Our study provides a methodologic framework to evaluate observational longitudinal microbiota data with exposure crossovers. We found COCs are associated with vaginal microbiota stability and a *Lactobacillus*-dominated state. COCs appear to impact stability within a month of initiation. Our findings have clinical implications for how soon benefits can be expected in (at least White) patients initiating COCs, and support the need for larger prospective trials to verify our results in ethnically diverse populations.

**Funding:**

R01-AI089878.

**Research in context**

**Evidence before this study**

Bacterial vaginosis (BV) is a common clinical condition in which the vaginal microbiome (the communities of bacteria colonizing the vagina) is characterized by low abundance of healthy lactobacilli and dominance by a variety of strict and facultative anaerobic bacteria. BV, which can result in vulvovaginal symptoms and an increased risk of poor reproductive and obstetric outcomes, is difficult to treat, with over 50% of patients experiencing recurrence within 6 months after antibiotic treatment. Therefore, identifying non-antibiotic treatments which help shift and/or stabilize the vaginal microbiota into a healthier, *Lactobacillus*-dominated state may help mitigate serious sequelae. Combined estrogen and progestin oral contraceptive pills (COCs) may be one such factor. Epidemiological studies have demonstrated a reduced risk of clinically-defined BV associated with use of hormonal contraception (HC), including COCs. Yet, to date, most studies have been small, cross-sectional or had very limited follow-up time. Few have used modern molecular techniques to define BV. Overall, little is known about whether, and in what time frame, COCs may act to affect the composition and stability of the vaginal microbiota.

**Added value of this study**

In the present study, we assessed the relationship of COCs and the vaginal microbiota through 16S rRNA gene amplicon sequencing of frequently-collected vaginal samples in a 2-year longitudinal observational study of individuals starting and stopping HC. This study is the first to describe the vaginal microbiota with such frequent sampling over such a prolonged period of time. COC users were more likely to have *Lactobacillus*-dominated microbiota and more stable microbiota over time. Stability increased and then plateaued four weeks after COC initiation. The associations between COCs and *Lactobacillus*-dominance, as well as microbiota stability, were statistically significant for White, but not for African American participants, possibly due to a smaller sample size in the latter group. Findings were similar to those for COC users when including other forms of HC and when excluding samples collected during menses.

**Implications of all the available evidence**

Our study provides a methodologic framework to evaluate complex observational longitudinal microbiota data. Our findings, that COCs are associated with vaginal microbiota stability and a *Lactobacillus*-dominated state, provide data to support future clinical trials to study the beneficial impact of COCs on the vaginal microbiota in patients with recurrent BV. Additionally, our findings may have immediate clinical implications since some clinicians use HC to help manage patients with recurrent BV. The longitudinal data suggest that any benefit in stabilizing the vaginal microbiota might be anticipated to occur within the first month after initiating HC. For patients in whom clinical improvement is not observed relatively quickly, other measures will likely be necessary to control recurrent episodes.

## Introduction

The vaginal microbiota play a critical role in supporting urogenital and reproductive health.^1^, ^2^, ^3^, ^4^, ^5^, ^6^, ^7^, ^8^, ^9^ The most protective, “optimal” vaginal microbiota are broadly characterized by a predominance of lactic acid producing *Lactobacillus* spp.^5^ The clinical condition of bacterial vaginosis (BV) is characterized microbiologically by low abundance of *Lactobacillus* spp., and dominance by a variety of strict and facultative anaerobic bacteria.^3^^,^^5^ Non-optimal BV states have been significantly associated with vulvovaginal symptoms and morbidity,^10^^,^^11^ as well as increased risk for sexually transmitted infections (STIs) including HIV,^1^^,^^3^^,^^4^ urinary tract infections (UTIs),^7^^,^^12^ miscarriage^13^ and preterm birth.^14^^,^^15^ Multiple factors, including sex hormones, menses, intravaginal hygiene practices and sexual practices, may adversely tip the composition and structure of the vaginal microbiota towards BV.^16^, ^17^, ^18^, ^19^, ^20^, ^21^, ^22^, ^23^ Because BV is difficult to treat, with over 50% of patients experiencing recurrence within 6 months after antibiotic treatment,^24^^,^^25^ identifying non-antimicrobial interventions which help shift and/or stabilize the vaginal microbiota in a *Lactobacillus*-dominated state may help mitigate serious sequelae. Combined estrogen and progestin oral contraceptive pills (COCs) may be one such intervention.

Multiple (mostly cross-sectional) epidemiological studies have demonstrated a reduced risk of BV, defined clinically [by Amsel's criteria^26^] or by Gram's stain [Nugent score^27^^,^^28^], associated with the use of hormonal contraception (HC), including COCs.^29^, ^30^, ^31^ These data suggest that HC may have a beneficial impact on the vaginal microbiota. A single pilot randomized controlled trial with a small sample size (N = 26 in the intervention arm), however, did not demonstrate a decrease in the incidence of recurrent BV after initiation of COCs.^32^ Several studies have used molecular approaches, such as 16S rRNA gene amplicon sequencing or qPCR, in addition to Gram's stain or clinical criteria, to characterize the impact of HC on the composition and structure of the vaginal microbiota. However, these studies have been small,^33^ cross-sectional,^34^, ^35^, ^36^, ^37^, ^38^ or have focused on HC methods other than COCs.^39^, ^40^, ^41^, ^42^, ^43^, ^44^, ^45^, ^46^, ^47^, ^48^ A single study of 40 adolescents examined the vaginal microbiota prior to and 16 weeks after initiation of COCs.^49^ The study demonstrated increased lactobacilli in the vaginal microbiota of COC users when comparing two time points over 16 weeks. Overall, there are few studies on whether, and in what time frame, COCs may act to affect the composition and stability of the vaginal microbiota.

COCs are among the most popular forms of HC worldwide and are often used over many years of an individual's reproductive life.^36^ Evaluating the impact of COCs on the vaginal microbiota may help inform the decision to investigate these agents as a treatment modality for patients with recurrent BV. Defining the timing of any beneficial effects on the vaginal microbiota following the initiation of COCs will have important clinical implications and help design future clinical trials. COCs may stabilize the vaginal microbiota in a *Lactobacillus*-dominated state within three months of initiation and *Lactobacillus-*dominance may be durable, however this has not been previously studied. Therefore, we assessed the relationship of COCs and the vaginal microbiota through dense interval sampling utilizing 16S rRNA gene amplicon sequencing in a 2-year longitudinal observational study. We assessed the specific timeframe in which COCs appear to increase stability of the vaginal microbiota by utilizing new statistical approaches to model complex temporal dynamics of the microbiota.

## Methods

### Study design/study setting

**Fig. S1:**
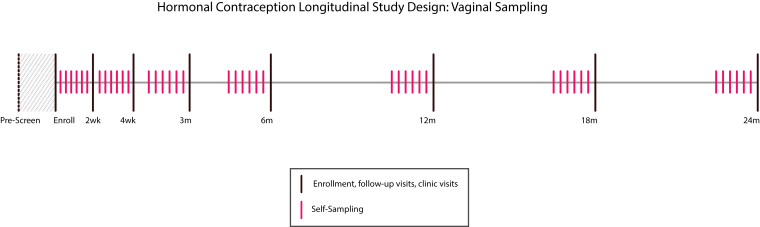


We analysed a subset (see sample selection, below) of midvaginal samples from the Hormonal Contraception Longitudinal Study, a cohort of reproductive-aged cisgender women in Baltimore, Maryland. Participants were recruited from outpatient Obstetrics and Gynecology clinics at a single institution from summer 2011-winter 2015. Inclusion criteria included reproductive age and self-reported intention to initiate or cease HC as well as recruitment of non-users who planned to avoid HC for the study period.^50^, ^51^, ^52^ Those with known pregnancy, hysterectomy, IUD, immunosuppression (such as from HIV or diabetes), conditions which might alter sex hormones (such as polycystic ovarian syndrome or premature ovarian failure), or contraindications to HC use were excluded. Participants filled out detailed symptom, demographic and behavioural surveys and underwent pelvic examinations with clinician-collected vaginal samples at enrollment and 7 follow-up visits (at 2 weeks, 4 weeks, and 3, 6, 12, 18 and 24 months) over two years (Supplementary Fig. S1). Additionally, participants filled out behavioural and symptom diaries, and self-collected vaginal swabs every other day in the two-week period before each visit. All vaginal samples were collected using E-swabs and placed in 1 ml of Amies transport medium (Copan Diagnostics, Murrieta, CA). Clinician-collected samples were immediately frozen at −80 °C, while self-collected swabs were stored in the participants’ home freezers and then transported with a cold-pack to the next study visit, where they were frozen at −80 °C. Prior work has demonstrated that self-collected swabs of the mid-vagina reflect similar bacterial composition as physician-collected swabs by culture-independent methodologies.^53^^,^^54^ Patients initiating or changing HC methods within 4 weeks of study initiation underwent additional vaginal sampling following that change with a frequency that paralleled the sampling frequency conducted during the first 4 weeks of study initiation.

### DNA extraction, sequencing and CST assignment of vaginal microbiota

DNA was extracted from vaginal E-swabs samples with either the QS DSP Virus/Pathogen Midi Kit (Qiagen) on the QiaSymphony platform or with the MagAttract PowerMicrobiome DNA/RNA Kit (Qiagen) using a custom automated protocol on a Hamilton STAR instrument, the latter if the samples performed poorly (<15,000 sequence reads) with the first round of sequencing. For the QiaSymphony kit, samples were thawed on ice and a 500 μl aliquot of cells suspension was used as input following the protocol described in Holm et al.^55^ For the MagAttract kit, samples were thawed on ice and a 200 μl aliquot of cells suspension was used as input following the manufacturer protocol. Prior to DNA extraction, cells were first lysed by bead-beating on a TissueLyser (Qiagen) at 20Hz for 20 min and the final elution volume was 110 μl. Negative controls of distilled sterile water were extracted in the same manner as samples.

**Fig. S2:**
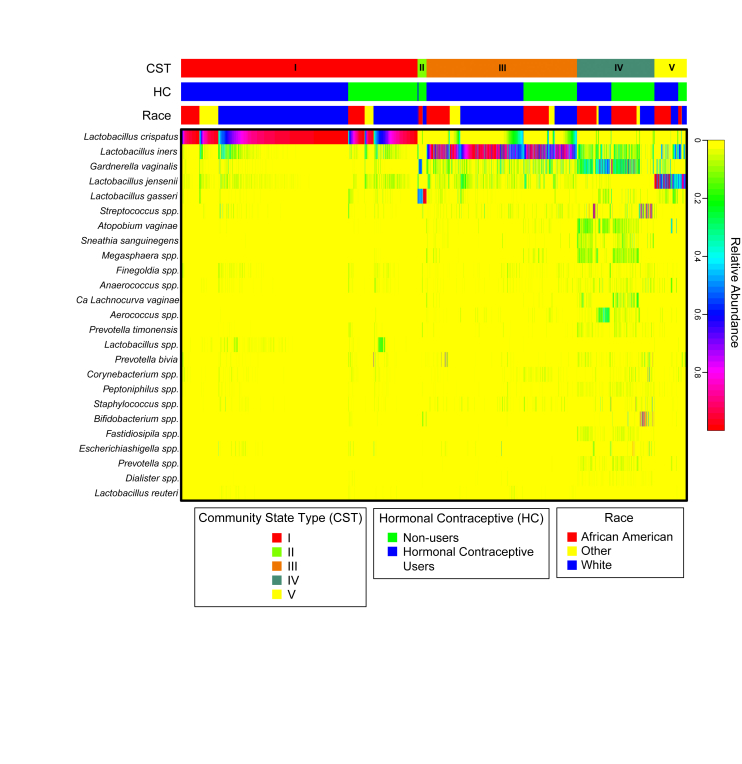


The composition of vaginal microbiota was established by sequencing amplicons of the V3–V4 region of the 16S rRNA gene. Amplification from DNA and library construction were performed with a 2-step PCR protocol and dual-barcoding strategy, and sequencing was carried out on the Illumina HiSeq 2500 platform with PE300 chemistry.^56^ The raw sequence data was processed using DADA2,^57^ and amplicon sequence variants (ASVs) were classified taxonomically at the genus level using the RDP Naïve Bayesian Classifier^58^ trained with the SILVA v128 16S rRNA gene sequence database.^55^^,^^59^ ASVs of major vaginal taxa were further speciated using speciateIT (http://ravel-lab.org/speciateit/). Community State Types (CSTs) were subsequently assigned using VALENCIA, a nearest centroid classification tool specifically designed for vaginal microbiota.^60^ CSTs were dominated by the following organisms: CST I – *Lactobacillus* *crispatus*, CST II – *Lactobacillus* *gasseri*, CST III – *Lactobacillus* *iners*, CST IV – diverse anaerobes, CST V *–* *Lactobacillus* *jensenii* (Supplementary Fig. S2).

### Sample selection

HC users were defined as participants using, stopping or starting any form of HC during the study, while non-users were defined as those who did not take HC for the duration of the study. For most analyses, we focused solely on COC users as they represented the largest proportion of HC modality in our study; however, as this was an observational study, all analyses were also performed with samples from all HC users. Participants who used progestin-only pills (POP) were excluded from the COC analyses. To ensure sufficient samples to enable meaningful longitudinal comparisons and stability estimates, participants with fewer than 10 longitudinal samples were removed from analyses. Since metronidazole use is known to impact the vaginal microbiota, vaginal samples taken within 2 weeks of oral or intravaginal metronidazole administration (in both HC users and non-users, N = 145 samples) were excluded. Additionally, samples from time intervals in which participants were not on HC (i.e., before starting or after stopping) or samples taken during the initial 3 months after HC initiation (i.e., during the “washout period”) were removed for most analyses (N = 826 samples). However, we conducted additional analyses utilizing samples from all available time points after HC initiation to define the time frame during which increased stability of the vaginal microbiota developed.

### Statistical analyses

We began by broadly determining if there were more *Lactobacillus*-dominated CSTs in the HC use vs non-use periods. Bayesian logistic regression mixed effects models were used to estimate statistical significance of differences in proportions of *Lactobacillus*-dominated and non-*Lactobacillus*-dominated CSTs (termed molecular-BV(4)) between COC user and non-user visits, as well as all HC users versus non-users, accounting for repeated measures within the same individual. HC status with or without stratification by race was a fixed effect and subject IDs were random effects. We also assessed statistical significance of differences in proportions of non-collapsed CSTs (I, II, III, IV and V) between COC user and non-user visits, as well as all HC users versus non-users, with and without stratification by race, also performing these analyses with menses samples removed.

We then measured vaginal microbiota stability during the 2-year follow-up with two analyses: A) Jensen-Shannon Index: a community level approach which measured the median Jensen-Shannon distance of each sample in a participant's longitudinal set from the participant's own centroid (the central measures of all CSTs within that individual)^61^ and B) CST evenness of the vaginal microbiota over time. For (A) Bayesian double exponential random effects models were used to estimated differences between the medians of participants' vaginal microbiota stability indices (measured Jensen-Shannon Index) within COC users and non-users participants as well as within all HC user and non-user participants. For (B) logistic regression models were used to estimate the dependence between the probability of HC use incidence and CST evenness, defined as,

$$\frac{-1}{\log\left(n\right)}\sum_{i=1}^{n}\log\left(p_{i}\right)p_{i}\;\text{,}$$

where p_i_ is the proportion of the i-th CST of the community and n is the number of CSTs (n = 5). A participant with a vaginal microbiota that stays in the same CST over time has a CST evenness of 0, as then p = 1, and log(1) = 0. On the other hand, a participant who has every CST with each at the same frequency over time, has p_i_ = 1/5 for every CST and hence the CST evenness is 1.

Finally, to determine the specific time frame after HC initiation over which vaginal microbiota stability increased or developed, we selected a sequence of time intervals (1–13 weeks) starting at the initiation of HC and compared stability index, S_*i*_, of a given participant's vaginal microbiota at the initial interval I, to the stability index, S_*p*_, of that individual's vaginal microbiota in the first dense sampling interval after interval I containing at least 3 samples. The stability index was defined as the median Jensen-Shannon distance from the centroid of the samples for which the index was computed (see Supplemental Methods for additional details). For each initial interval I, the mean of log ratio log_10_(S_*i*_/S_*p*_) was computed with 95% confidence intervals estimated using t-test. The value of the log ratio log_10_(S_i_/S_p_) is negative if the stability of a given participant's microbiota at the first dense sampling interval after interval, I, is higher than the stability of that participant's microbiota during interval I. For the given time interval, I, starting at the initiation of HC, the mean of log ratios over all participants quantifies the overall difference in stability of the vaginal microbiota between the interval I and the first dense sampling interval after interval I. The first interval I with the mean log_10_(S_*i*_/S_*p*_) whose 95% confidence intervals did not contain 0, was defined as the time from HC initiation after which significantly increased vaginal microbiota stability developed. All Bayesian models were implemented using the R package rstan, which is the R interface to the Stan - statistical modelling and high-performance statistical computation platform [S, rS]^62^^,^^63^ (see Supplemental Methods for additional details). All analyses were carried out using R package [R Core Team (2020). R: A language and environment for statistical computing. R Foundation for Statistical Computing, Vienna, Austria. URL https://www.R-project.org/], or Stata v17 (College Station, TX).

#### Sample size estimation

This was a convenience sample based on utilizing all available samples from patients recruited to the parent study not meeting exclusion criteria (see above).

#### Replicates

For the 16S rRNA gene amplicon sequencing, positive and negative controls were run alongside samples on each plate. Positive extraction controls contained an in-house mock community using isolates from the vaginal microbiome. Positive PCR controls were the Zymobiomics Microbial Community DNA Standard (Zymo Research). PCR and extraction negative controls contained distilled sterile water.

#### Randomization/blinding

Not Applicable.

#### Ethics statement

This study was approved by the Johns Hopkins Institutional Review Board (NA00043112) and the University of Maryland Baltimore Human Research Protections Office (HP-00045732). Informed consent was obtained from all participants.

### Financial disclosure/role of the funding source

This research was supported by 10.13039/100000002NIH grant R01-AI089878 (PI: Ghanem). ST is supported by 10.13039/100000002NIH grant K23-AI125715. The funder had no role in study design, data collection and analysis, decision to publish, or preparation of the manuscript.

## Results

**Fig. 1:**
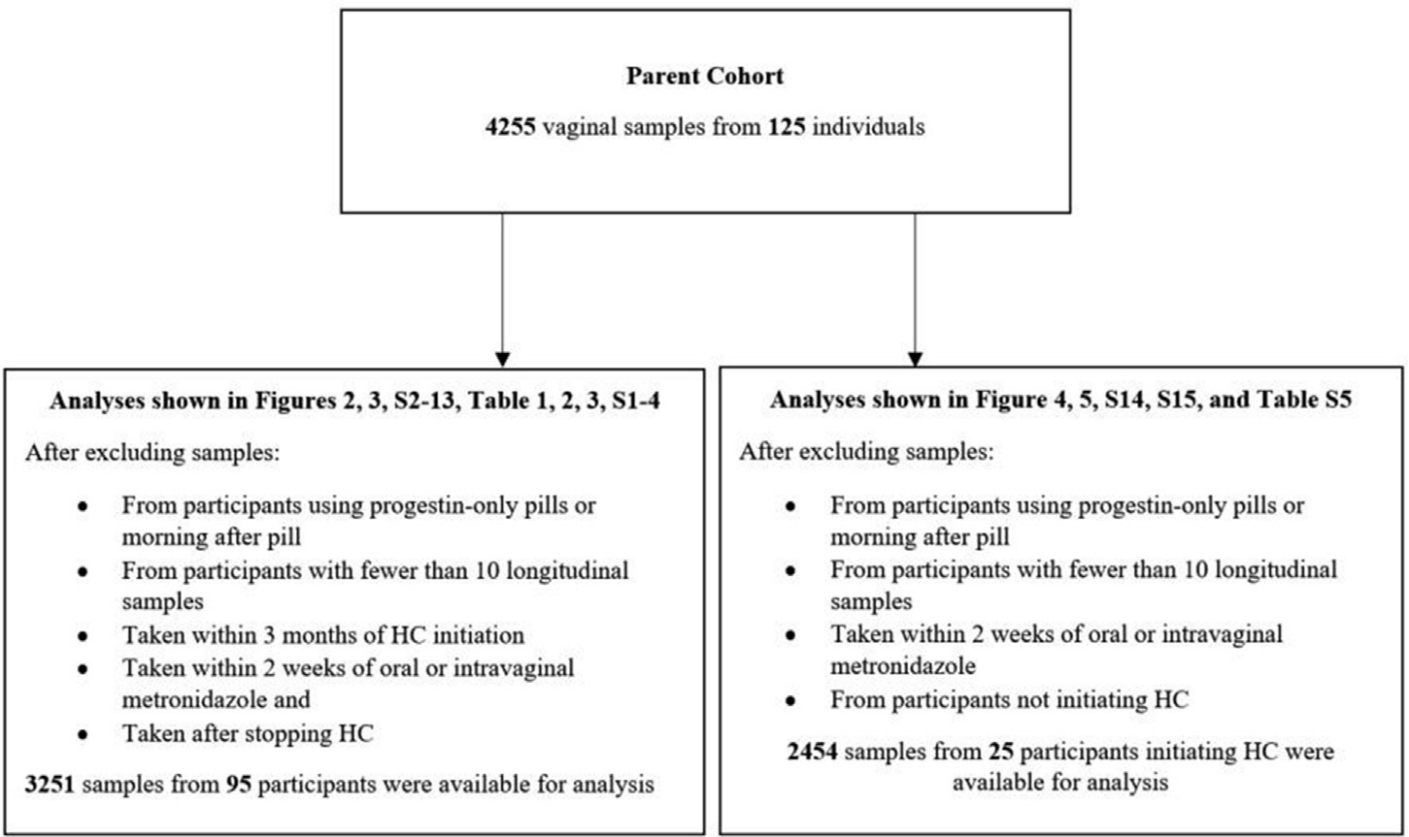
Flow diagram of samples and subjects.

After exclusions, 3251 samples from 63 participants on HC (including 16 African American and 40 White) and 32 participants never on HC in the course of the study (including 14 African American and 15 White) were available for initial analysis (Fig. 1). All participants answered survey questions reported in Table 1. Overall, only 1.3% of samples were missing 16S rRNA gene amplicon sequencing data due to either poor quality of extracted DNA or not sufficient 16S rRNA gene sequence count (both random events). Of the HC users, 46 used oral contraceptive pills (OCPs) (including N = 35 White, N = 7 African American and N = 4 participants of other ethnicities), the remainder used a combination of vaginal rings, implants, medroxyprogesterone acetate injections, patches or hormonal IUDs. All OCP users used estrogen and progestin combination oral contraceptive pills (COCs). Users and non-users were similar in terms of race, number of recent sexual partners, condom use and douching practices (Table 1). For most of the analyses presented below, we focused on analysis utilizing samples from COC users (46 participants) and non-users (32 participants), with a total of 1413 samples from COC users and 1174 samples from non-users. When comparing COC users to non-users at baseline, COC users were more likely to be White (76.1% vs. 15.2%, p = 0.02 [chi-squared test]), and were slightly younger (mean age 24.3 vs. 26.8, p = 0.01 [t-test]); all other factors were not statistically significantly different. Only one (white) woman in the control group used a copper IUD.

**Table 1:** Baseline (enrollment) characteristics of HC users and non-users.

	Overall N = 95	HC users N = 63	Non-users N = 32
**Age (median, interquartile range)**	25.8 (21.9–29.5)	25.1 (21.8–28.4)	26.9 (22.2–31.0)
**Race**			
White	55 (57.9)	40 (63.4)	15 (46.9)
African American	30 (31.6)	16 (25.4)	14 (43.8)
Other	10 (10.5)	7 (11.1)	3 (9.4)
**Number of male sexual partners in last 2 months**			
0	15 (15.8)	9 (14.3)	6 (18.8)
1	76 (80.0)	52 (82.5)	24 (75.0)
≥2	4 (4.2)	2 (3.2)	2 (6.3)
**Number of female sexual partners in last 2 months**			
0	93 (97.9)	62 (98.4)	31 (96.9)
1	2 (2.1)	1 (1.6)	1 (3.1)
≥2	N/A	N/A	N/A
**Condom use with vaginal sex in last 2 months**			
Never	38 (40.0)	28 (44.4)	10 (31.3)
Sometimes	12 (12.6)	6 (9.5)	6 (18.8)
Always	33 (34.7)	21 (33.3)	12 (37.5)
N/A	12 (12.6)	8 (12.7)	4 (12.5)
**Types of HC used∗**	N/A		N/A
COC		46	
Patch		2	
Ring		6	
Injection		4	
Implant		5	
Hormonal IUD		3	
**Douching in last 2 months**			
≥Once per month	3 (3.2)	1 (1.6)	2 (6.3)
≥Once in 2 months	1 (1.1)	1 (1.6)	0 (0.0)
None	91 (95.8)	61 (96.8)	30 (93.8)
**Educational attainment**			
HS or some HS	8 (8.4)	4 (6.4)	4 (12.5)
College or some College∗∗	58 (61.1)	37 (58.7)	21 (65.6)
Grad school or some Grad school	29 (30.5)	22 (34.9)	7 (21.9)

**Fig. S3:**
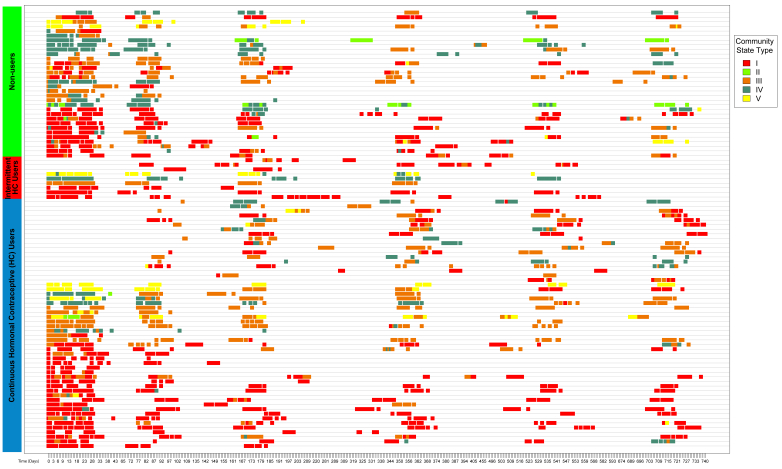


P value for age was 0.06. For all other relevant comparisons p value was >0.2 [Chi-squared tests were utilized except for age variable where t-test was utilized]. ∗During entire study. ∗∗Including community college. Note that a few patients switched HC types (see Supplementary Fig. S3), HC = hormonal contraception, COC = combined oral contraceptive pill, IUD = intrauterine device, Injection = Medroxyprogesterone acetate shot. All implant users used either nexplanon or implanon. HS = high school, Grad = graduate.

### Vaginal community state (CST) profiles over time: COC users versus non-users

**Fig. 2:**
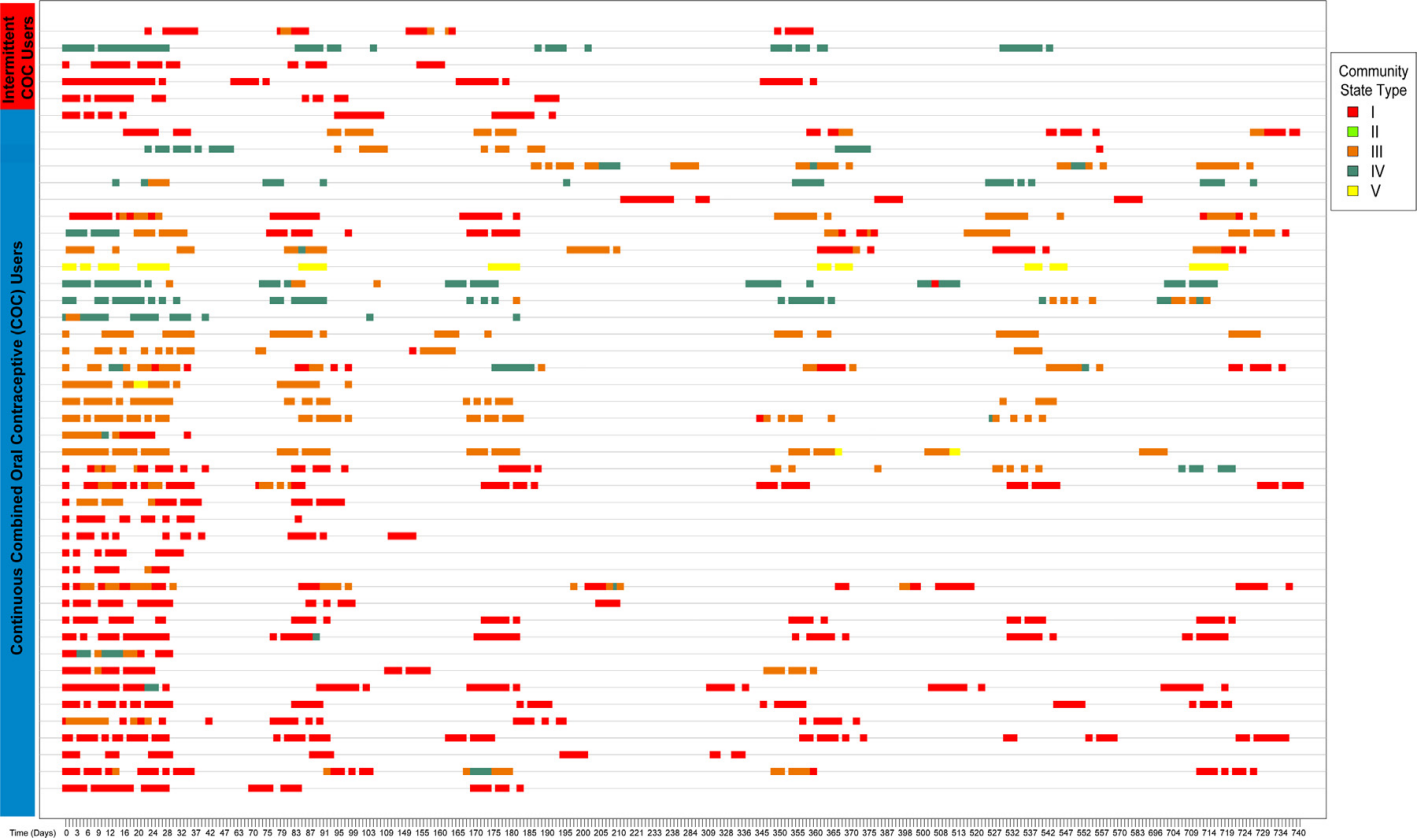
Vaginal community state type profiles over time: combined estrogen and progestin oral contraceptive pill users in the paper. Vaginal community state types over time in N = 46 participants included in the study who used combined estrogen and progestin oral contraceptive pills (COC) with either continuous or intermittent use. Community state type (CST) I is dominated by *L. crispatus*, CST II by *L. gasseri*, CST III by *L. iners*, CST V by *L. jensenii*, and CST IV is low in *Lactobacillus* spp. and dominated by diverse anaerobes.

**Fig. S4:**
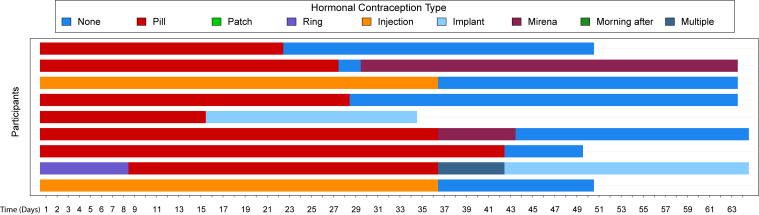


Overall, the vaginal microbiota of most participants (whether HC users or non-users) was stable over two years (see Fig. 2 for CST profiles of COC users and Supplementary Fig. S3 for all HC users and non-users). Nine participants (listed in Supplementary Fig. S3 as “intermittent HC”) stopped or switched forms of HC (Supplementary Fig. S4), however, samples collected after stopping or switching HC were not included in our analyses below.

Utilizing a Bayesian logistic regression mixed effect model with participant-level random intercept, accounting for repeated measures within the same individual, the proportion of CST IV (i.e. low-*Lactobacillus*, “molecular-BV”) assignments over the follow-up period samples of COC users was lower than the proportion of molecular-BV assignments among non-users (Table 2). The proportion of CST I (*L. crispatus*-dominated) over the follow-up period samples of COC users was higher than the proportion of CST I among non-users (Table 3). *L. crispatus*-dominated communities are significantly positively associated with COC based on our analyses. When samples taken at the time of menses were removed from analyses (Supplementary Tables S1 and S2), results were unchanged. Decreases in proportions of CST IV vs. *Lactobacillus*-dominated CSTs (CST I, II. III and V) in non-users were statistically significant in White but not in African American participants (Supplementary Tables S1 and S2). The sample size was much smaller when the analysis was restricted to African American participants (N = 7 African American COC users vs. N = 14 African American non-users) as compared to White participants (N = 35 White COC users vs. N = 15 White non-users). Further, results were similar when samples from all HC users were included (Supplementary Tables S3 and S4).

**Table 2:** Proportions of samples from COC users versus non-users in *Lactobacillus*-dominated vs. non-*Lactobacillus*-dominated vaginal Community State Types.

	Non-*Lactobacillus* CSTs (i.e. CST IV) N (%)	*Lactobacillus* CSTs N (%)
COC users	150 (10.6)	1263 (89.4)
Non-users	277 (23.6)	897 (76.4)
Point estimates and CrIs	−2.7 (−4.56,-1.03)	0.099 (0.02,0.27)

N = number of samples, Non-*Lactobacillus* CST = non-*Lactobacillus* dominated Community State Type; i.e., CST IV, *Lactobacillus* CSTs = *Lactobacillus-*dominated Community State Types, non-users = samples from those who never used HC during the entire study, COC = combined estrogen and progestin oral contraceptive pill. Multiple samples per participant are included in this table. Point estimates and the corresponding credible intervals (CrIs) were estimated using Bayesian mixed effects Bernoulli models (see Methods – Supplemental Materials for details) with subject-wise random intercept. The point estimates are the log ratios of the estimated proportions of the given CST between COC users and non-users. As such they are not the log ratios of the sample proportions of the CSTs within COC users and non-users.

**Table 3:** Proportions of Samples from COC users versus non-users in each vaginal Community State Type.

	CST I N (%)	CST II N (%)	CST III N (%)	CST IV N (%)	CST V N (%)
**COC users**	794 (56.2)	5 (0.4)	380 (26.9)	150 (10.6)	84 (5.9)
**Non-users**	446 (38.0)	52 (4.4)	344 (29.3)	277 (23.6)	55 (4.7)
Point estimates and CrIs	2.7 (0.75,5)	−3.4 (−8,0.41)	−0.9 (−2.2,0.4)	−2.7 (−4.5,−0.96)	−0.59 (−4.1,2.7)

N = number of samples, Non-users = samples from those who never used HC during the entire study, CST = community state type, COC = combined estrogen and progestin oral contraceptive pill. Point estimates and the corresponding credible intervals (CrIs) were estimated using Bayesian mixed effects Bernoulli models (see Methods – Supplemental Materials for details) with subject-wise random intercept. The point estimates are the log ratios of the estimated proportions of the given CST between COC users and non-users. As such they are not the log ratios of the sample proportions of the CSTs within COC users and non-users.

### Vaginal microbiota stability over time: COC users versus non-users

**Fig. 3:**
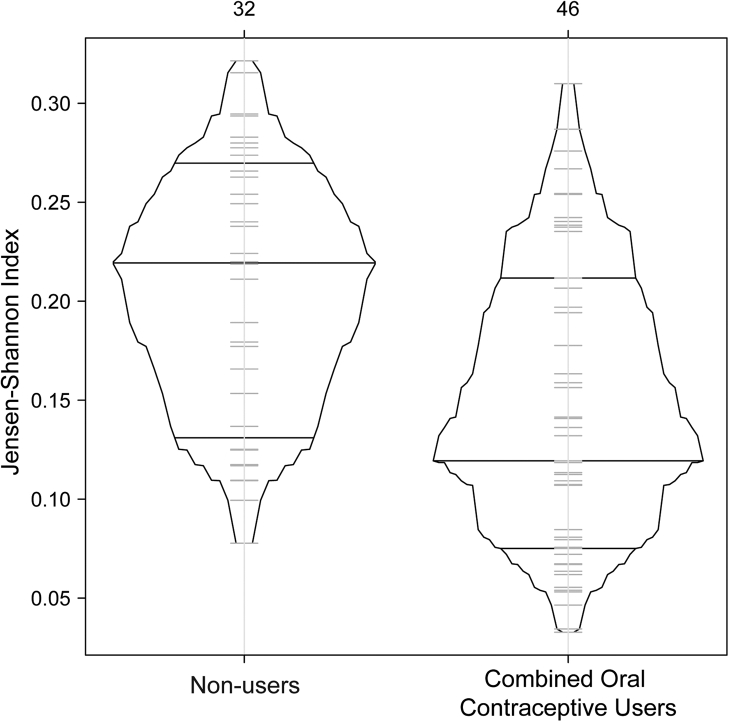
Vaginal microbiota stability over two years: box-percentage plot of Jensen-Shannon indices in samples from COC users versus non-users. Combined estrogen and progestin oral contraceptive pill users (COC) users (N = 1174 samples from 46 participants) had increased vaginal microbiota stability as compared to non-users (N = 1413 samples from 32 participants). Median Jensen-Shannon indices in COC users: 0.22 versus 0.13 in non-users, the difference: −0.09, CrI: (−0.13,-0.045).

**Fig. S5:**
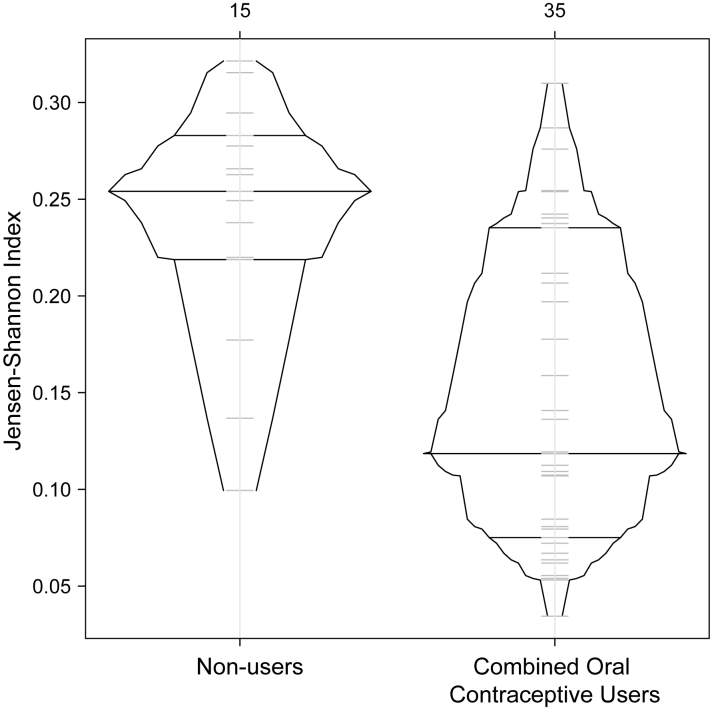


**Fig. S6:**
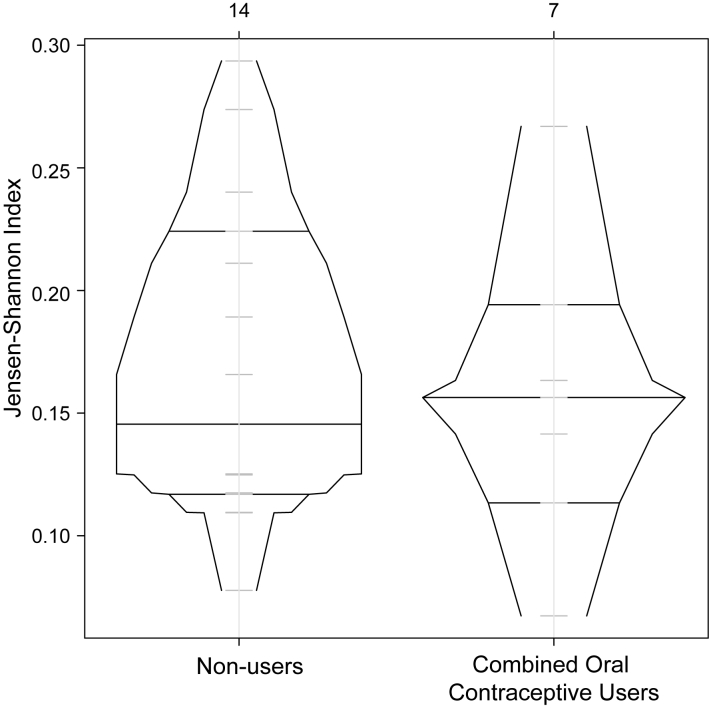


**Fig. S7:**
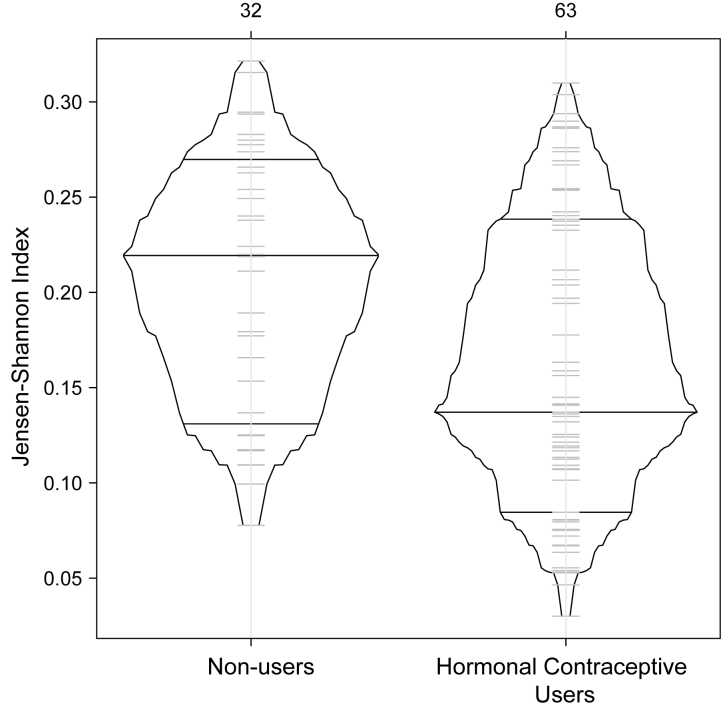


**Fig. S8:**
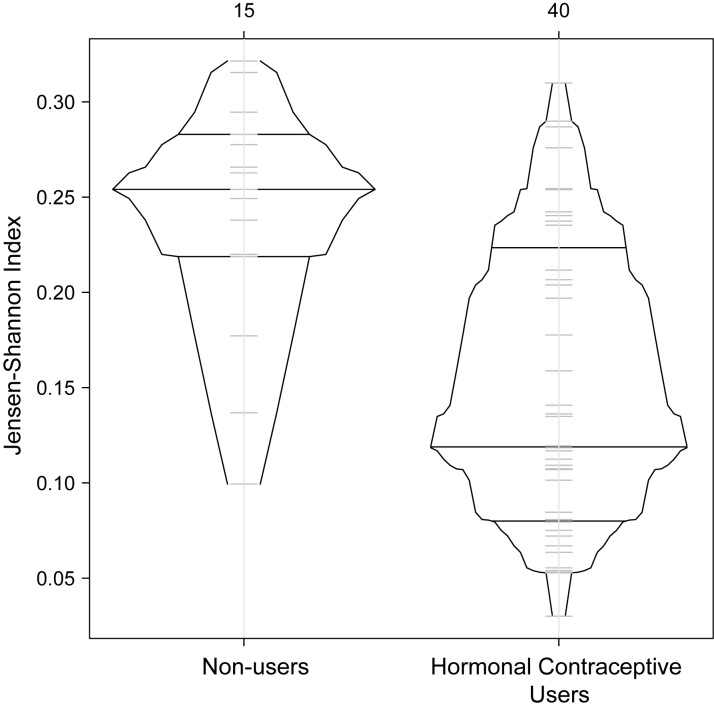


**Fig. S9:**
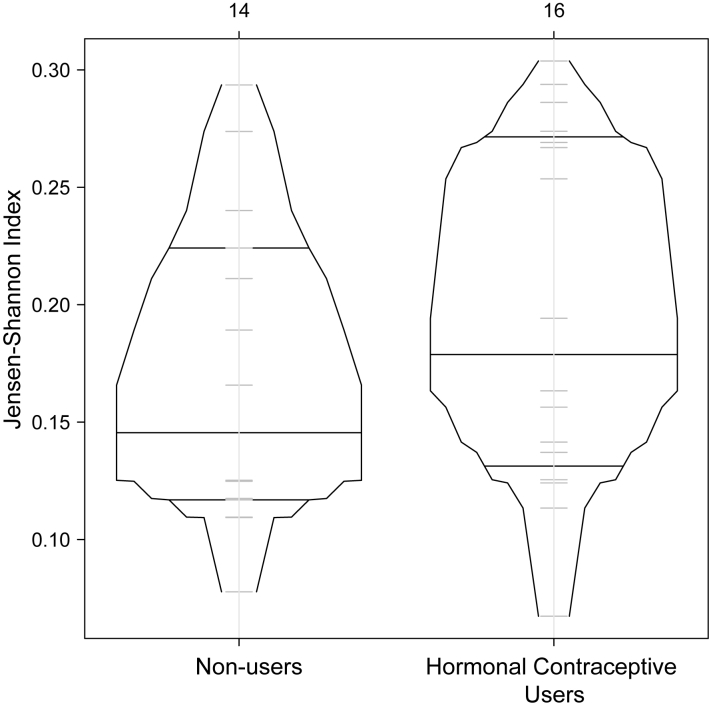


When comparing stability after HC initiation, COC users demonstrated higher stability compared to non-users who never used HC during the entire study when evaluated by Jensen-Shannon Index (Fig. 3). This relationship was statistically significant for White but not for Black participants (Supplementary Figs. S5 and S6). Similar results were observed when samples from all HC users were included (Supplementary Figs S7–9).

**Fig. S10:**
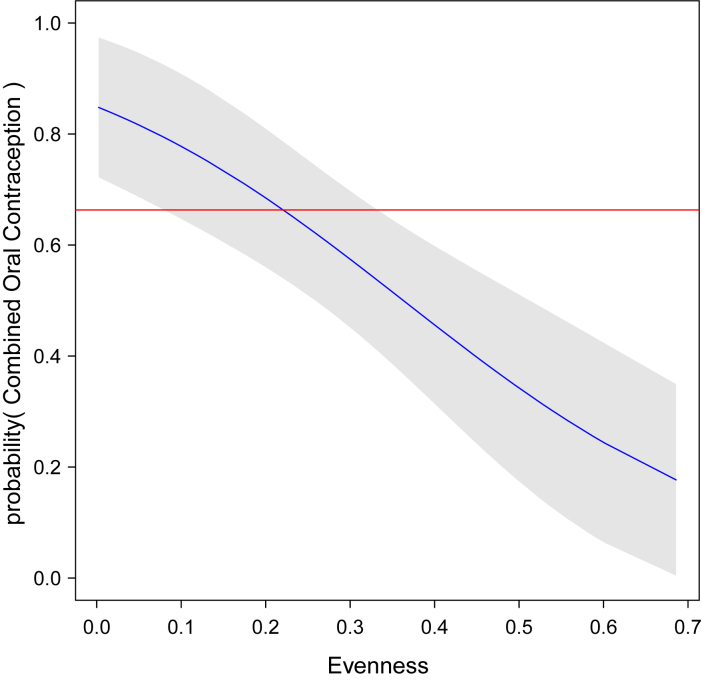


**Fig. S11:**
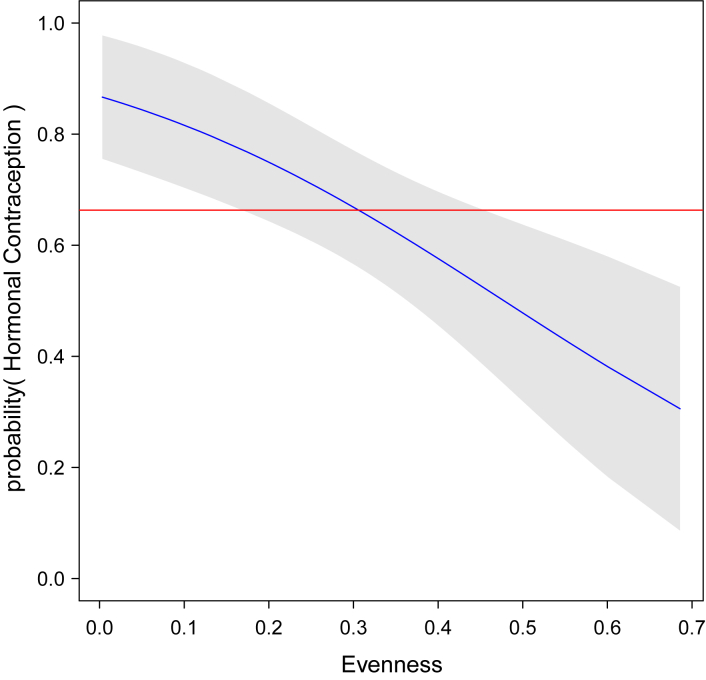


**Fig. S12:**
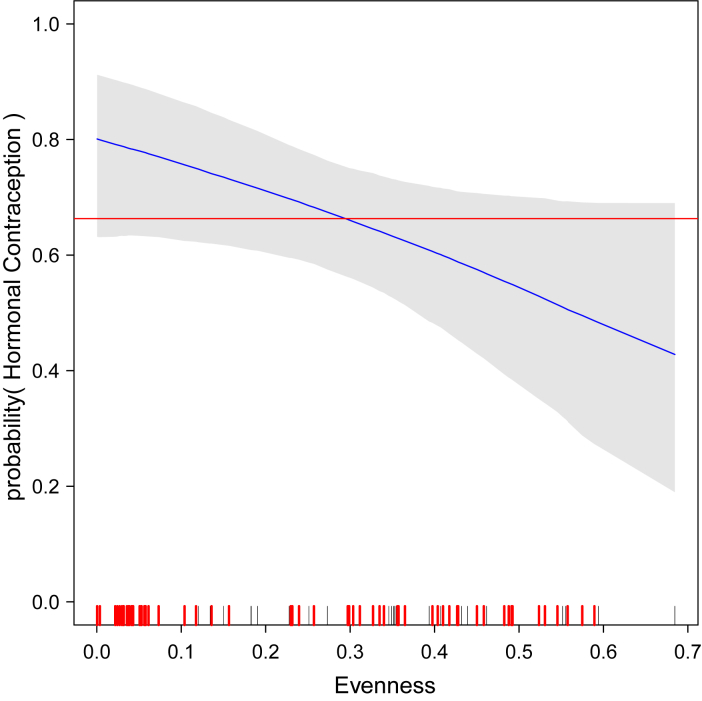


**Fig. S13:**
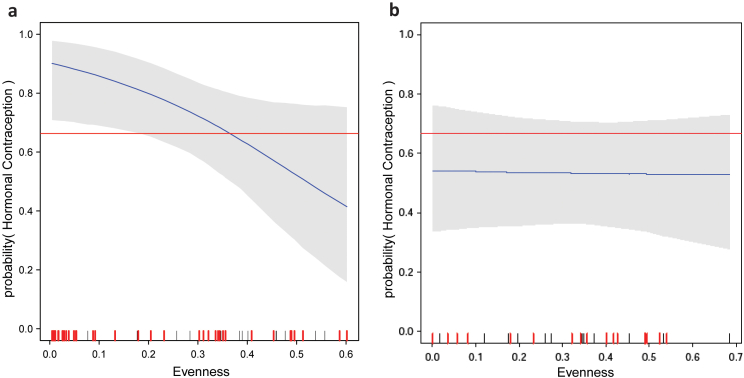


Similarly, using CST evenness to measure vaginal microbiota stability, COC users had more stable vaginal microbiota compared to non-users not on HC (Supplementary Fig. S10). The same finding was observed when all HC users were included (Supplementary Fig. S11), even if samples collected during menses were removed from the analysis (Supplementary Fig. S12). Again, results were only statistically significant for White but not for African American women (Supplementary Fig. S13).

### Time frame after which vaginal microbiota stability establishes post-COC initiation

**Fig. 4:**
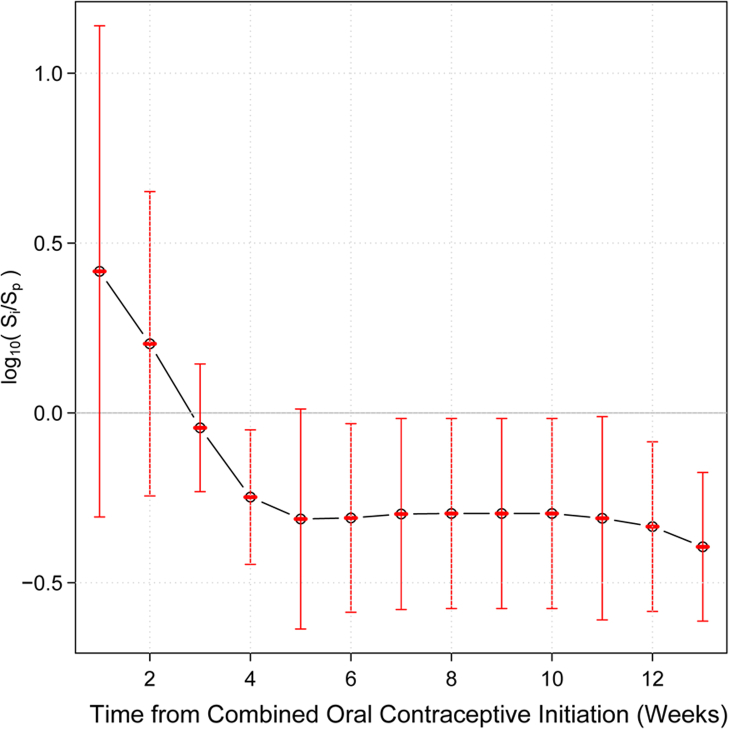
Timeframe after COC initiation in which vaginal microbiota stability increases. This plot shows the dependence between the mean of log ratios of the stability index and the length of time from the initiation of combined estrogen and progestin oral contraceptives (COCs). The stability of the vaginal microbiota in the first dense sampling interval after initiation of COC is significantly higher than during initial interval I on average after 4 weeks from the initiation of COCs. Four weeks after initiation, the stability of the vaginal microbiota remains constant. This analysis utilized 1590 samples from 18 participants.

**Fig. 5:**
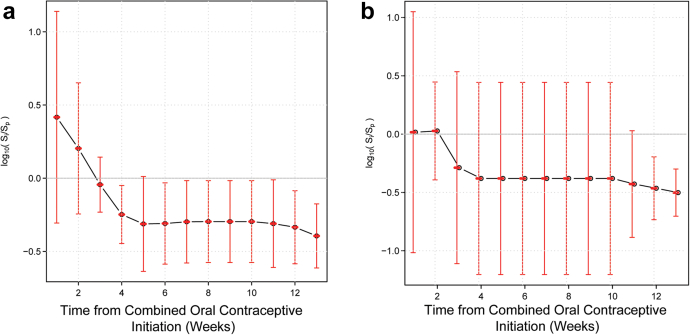
Timeframe after COC initiation in which vaginal microbiota stability increases in White (Panel a, left) and African American (Panel b, right) participants. Dependence between the mean of log ratios of the stability index and the length of time interval I, from the initiation of combined estrogen and progestin oral contraceptive pills (COCs) in White participants (N = 10) (Panel a, left) and African American participants (N = 4) (Panel b, right). The stability of the vaginal microbiota in the first dense sampling interval after initiation of COC is significantly higher than during initial interval I on average 4 weeks from initiation of COCs in White participants. Four weeks after initiation, the stability of the vaginal microbiota remains constant. A similar trend is seen in African American participants, but it is not statistically significant possibly due to small sample size.

**Fig. S14:**
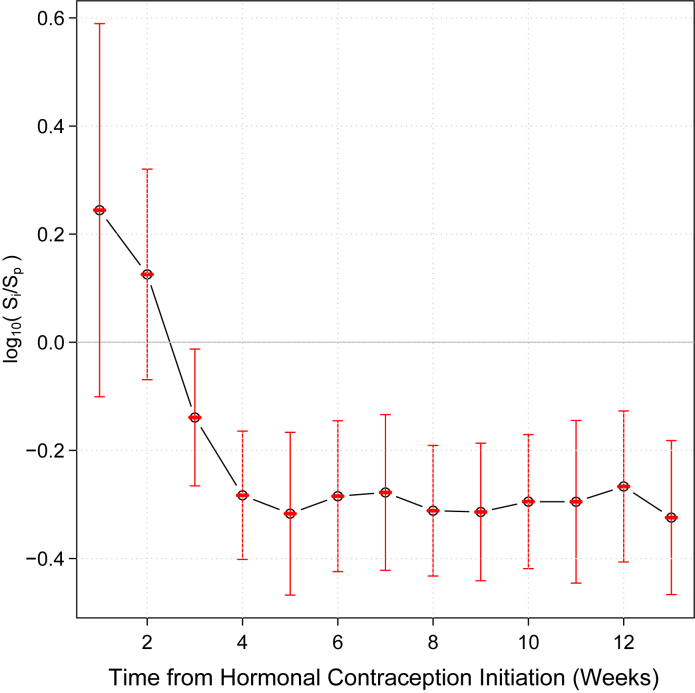


**Fig. S15:**
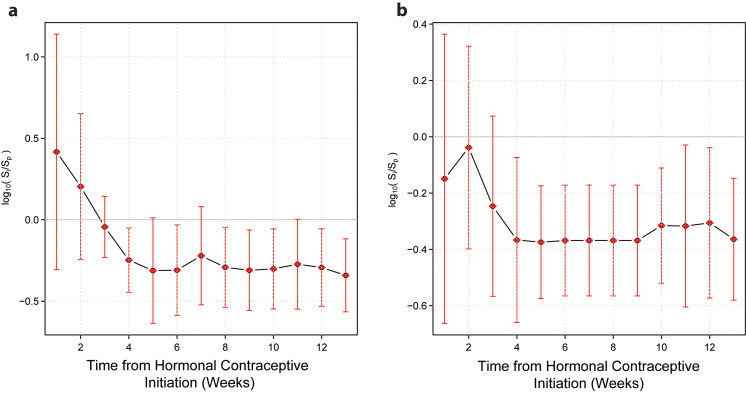


Finally, to ascertain the time frame necessary for the microbiota to stabilize after HC initiation, we analysed data from participants initiating HC. N = 2454 samples from N = 25 participants were available for analysis (Fig. 1). Five participants (4 initiating COCs and 1 initiating other forms of HC) who did not appear in Table 1 were included in this analysis. Baseline participant characteristics when including these additional participants did not differ significantly from the N = 95 included in the main analyses (Supplementary Table S5). We analysed samples from N = 10 White, N = 4 African American, and N = 4 participants of other ethnicities initiating COCs for a total of N = 18. We found that stability on average becomes statistically significantly higher after 4 weeks on COCs. After 4 weeks, no further increase in vaginal microbiota stability was observed (Fig. 4). Results were consistent in stratified analysis limited to White participants, and similar, though not statistically significant, trends were seen in stratified analysis limited to African American participants (Fig. 5). When utilizing samples from all participants initiating HC, stability increases appear statistically significant at 3 weeks and level off after 4 weeks (Supplementary Fig. S14). Results were similar in stratified analysis limited to White and Black participants initiating any HC (Supplementary Fig. S15). Stability appeared durable over at least 13 weeks while on COCs.

## Discussion

In a two-year observational study of the vaginal microbiota (the first to follow participants with such frequent vaginal sampling over such a prolonged period of time), we found that those on COCs were more likely to have *Lactobacillus*-dominated vaginal microbiota over time, and overall, had more stable vaginal microbiota than those who did not use any HC. The association between COCs, *Lactobacillus* dominance, and vaginal microbiota stability was statistically significant for White, but not for African American participants- but the latter demonstrated similar trends. This finding may be due to limited sample size among African American participants. Of note, after COC initiation, stability increased and then plateaued after about 4 weeks on COCs. This trend for increased stability was not statistically significant in African American participants. Results were similar when those utilizing any forms of HC were considered or when samples taken during menses were removed from the analyses.

Our results for COCs are consistent with multiple studies that demonstrated a reduced risk of incident and prevalent BV among those using combined estrogen and progestin contraceptive methods.^29^, ^30^, ^31^^,^^64^^,^^65^ Our results are also similar to other studies evaluating the effect of COCs on the composition of the vaginal microbiota characterized using molecular approaches (16S rRNA gene amplicon sequencing or qPCR), including a cross-sectional study (N = 682) comparing the vaginal microbiota of women on HC to those who used condoms for contraception.^34^ That study found that those using COCs (N = 206) were more likely to be colonized by *Lactobacillus* spp. compared to a condom-alone group (N = 186). Progestin-only depo-medroxyprogesterone acetate (DMPA) (N = 94) and levonorgestrel-releasing intrauterine system (LNG-IUS) (N = 196) users, however, were not more likely to be colonized by *Lactobacillus* spp. In a complex crossover trial in South Africa,^49^ a group of mostly Black adolescent girls were randomized to: 1) COCs (N = 40) and after 16 weeks switched to combined estrogen-progestin vaginal rings (CCVR); 2) CCVR (N = 45) and after 16 weeks switched to COCs or progestin-only injectable contraceptives (POC); and 3) POC (N = 45) and after 16 weeks switched to CCVR. Despite no significant differences in the vaginal microbiota composition or structure at baseline, the study found that at the 16-week crossover, there was a lower proportion of COC users with low-*Lactobacillus* vaginal microbiota as compared to POC users, with a non-statistically significant trend for similar differences between CCVR users and POC users. COC users had lower alpha diversity (i.e., fewer different kinds of bacteria present in the vaginal communities) as compared with CCVR and POC users.^49^

Studies evaluating combined estrogen and progestin contraceptives other than COCs have not yielded consistent results. Crucitti^45^ reported that initiation of combined estrogen-progestin in the form of intravaginal rings (N = 120) promoted vaginal *Lactobacillus* spp.^45^ There was a similar, though non-statistically significant trend in the study by Balle et al*.*^49^ A small study (N = 15) of individuals initiating CCVR while prophylactically being treated with tenofovir did not report any significant changes to the microbiota of CCVR recipients compared to recipients of a placebo ring.^46^ Finally, a study of 40 individuals using a combined estrogen and progestin injectable contraceptive did not find a significant impact of this method on the composition and structure of the vaginal microbiota when using qPCR to measure *Lactobacillus* spp., *Gardnerella vaginalis*, *Atopobium vaginae* and *Megasphaera phylotype 1*.^39^

While our study and others support COC use as a contributor to promoting *Lactobacillus* dominance and stability, emerging data suggest that progestin-only contraceptives (POCs) may not have the same beneficial effects.^65^ An early systemic review and meta-analysis^29^ found that POC (based on N = 6 studies) was associated with decreased prevalent and incident BV. However, of the six included studies reporting on prevalent BV, only two reported a statistically reduced prevalence, and of the six included studies reporting on incident BV, only one reported a statistically reduced incidence. Several more recent studies employing sequencing-based evaluation of the microbiota have found little to no impact of POC on the vaginal microbiota.^34^, ^35^, ^36^^,^^38^, ^39^, ^40^^,^^42^^,^^48^^,^^66^ While a study of N = 15 found no impact of POC initiation on the abundance of vaginal *Lactobacillus* spp., it reported decreases in the abundance of the BV-associated bacterium *G. vaginalis*.^42^ Several other studies have reported decreases in vaginal lactobacilli or increases in some BV-associated bacteria after initiation of POC.^33^^,^^41^^,^^43^^,^^44^^,^^47^ One of these studies found this possibly detrimental effect in African American, but not in Latina participants.^41^ To further corroborate this, a recent systematic review found that estrogen containing compounds overall seemed to promote an optimal vaginal microbiota, but the effect of progestin only contraceptives was much less clear, with some concerns for potential negative effects.^65^ In our study, when participants using all forms of HC were included, the results did not change, participants remained more likely to be stable and more likely to be dominated by *Lactobacillus* spp. if they were using HC. However, we had relatively few POC users in the study. Thus, our results including all HC users may have been driven by the predominant COC users.

The mechanisms by which COC use may promote stability and *Lactobacillus-*dominated vaginal microbiota are not known, but they are postulated to be mediated by estrogen. Estrogen increases glycogen production by vaginal epithelial cells, which in turn support the growth of vaginal lactobacilli.^67^ Thus, the estrogen component of COCs (lacking in POCs) may contribute to these effects. It is important to note, that estrogen is necessary but not sufficient to support growth of lactobacilli as many anaerobic bacteria can also use glycogen as carbon source.^68^, ^69^, ^70^ The strains of *Lactobacillus* spp. present might also play an important role in the establishment of a *Lactobacillus-*dominated vaginal microbiota after COC initiation. Menstruation has been shown to be associated with a low-*Lactobacillus* microbiota.^16^^,^^17^ It is hypothesized that blood provides a favourable environment for the growth of vaginal anaerobic bacteria. Thus, HC may inhibit uterine bleeding and reduce menstrual blood loss promoting *Lactobacillus-*dominated vaginal microbiota stability. However, our study suggests that the effect of HC on stabilization of the microbiota is not only mediated by its effects on decreasing menstruation - we found that removal of samples taken during the time of menses did not affect the relationship between COCs and vaginal microbiota stability or *Lactobacillus* dominance. Of note the effect on menstrual bleeding is predominantly seen with POCs, in which amenorrhea may result after prolonged use, rather than in COCs.^67^ However, there were relatively few samples collected during menstruation, so it is also possible we had insufficient power to observe an effect.

We did not observe a benefit to COC use in terms of promotion of vaginal *Lactobacillus* dominance and vaginal microbiota stability in African American participants. However, we observed a similar trend in the analysis of the time required to achieve stability post-HC initiation, suggesting that the lack of statistical significance may be related to the relatively small number of African American COC users in our study. This is an important question to answer because the prevalence of BV in African American women is two times that observed in White women.^71^ Larger studies of HC users with multiple ethnicities should elucidate whether ethnic differences exist. We attempted to address race/ethnicity by conducting stratified analyses, but small sample size results may not be fully generalizable to non-White populations.

This study had several additional limitations. The observational nature of this study may have introduced unmeasured biases with respect to which patients chose to start HC. The overall characteristics of the HC and non-user groups were balanced with respect to age, race, number of sexual partners, condom use, and douching practices-factors which have been shown to impact the vaginal microbiota, however we did not evaluate these factors longitudinally and cannot with certainty exclude all potential sources of unmeasured bias. We relied on self-report of both HC use and demographic and behavioural factors. Although all interviews were conducted in private rooms with a trained study coordinator and participants were assured of confidentiality, it is possible that some bias could have been introduced in this process, and we do not have serum hormone measurements to validate the self-reported HC use. COC users were slightly younger than non-users. All participants, however, were of reproductive age and the absolute difference in mean age between the two groups was less than 2 years (24.6 vs. 26.8 years), and unlikely to be of clinical significance. Since COC users were more likely to be White than African American, and since African American race is in previous literature so strongly associated with CST IV (i.e., Bacterial Vaginosis/molecular-BV) we did conduct stratified analysis in White and African American individuals to examine the influence of this factor. Because this was an observational study, participants chose to initiate or cease HC, and this study design resulted in a large proportion of our participants having few baseline samples prior to HC initiation. Statistically, this presented challenges for a quasi-experimental cross-over assessment of a participant prior to and following HC initiation or cessation. The use of Jensen-Shannon Index and CST evenness as measures of microbiota stability provided novel approaches to account for this limitation. Although subset analyses were limited due to small sample sizes, the dataset did offer a 2-year follow-up with over 4500 samples collected from 125 individuals. The observational nature of this study may have introduced unmeasured biases with respect to which patients chose to start HC, however, randomized trials of HC modalities are difficult to launch and recruit. The prevalence of BV within the reproductive-aged group has not been shown to vary by age.^71^ Finally, while we found that participants on HC were more likely to be *Lactobacillus*-dominated overall, and had increased stability after HC initiation, due to sample size, we were unable to assess the extent to which HC was able to shift participants who started in a low-*Lactobacillus* state to a *Lactobacillus*-dominated state after HC initiation.

In summary, our study provides a methodologic framework to evaluate observational longitudinal microbiota data and provides important insights into the impact of COCs in promoting vaginal microbiota health and stability, and the time frame in which this benefit occurs. Larger studies are needed to verify our findings in ethnically diverse populations. If results prove consistent, these will support the utility of a larger clinical trial to study the impact of COCs (either alone or as part of combination therapy with other interventions) in patients with recurrent BV. Currently, some clinicians use HC to help manage patients with recurrent BV. Our data, at least preliminarily, suggest that any benefit in stabilizing the vaginal microbiota might be anticipated to occur within the first month after initiating HC. If clinical improvement is not observed relatively quickly, other measures will likely be necessary to control recurrent episodes in this patient population.

## Contributors

Susan Tuddenham: Data curation, Formal Analysis, Writing - Original draft, review and editing; Pawel Gajer: Data curation, Formal Analysis, Software, Writing - Original draft, review and editing; Anne Burke: Methodology, Project Administration, Writing - review and editing; Catherine Murphy: Data curation, Investigation, Project Administration, Writing - review and editing; Sabra Klein: Conceptualization, Writing - review and editing; Christina Stennett: Visualization, Data curation, Writing - review and editing; Barbara Wilgus: Data curation, Investigation, Project Administration, Writing - review and editing; Jacques Ravel: Conceptualization, Methodology, Supervision, Writing - review and editing; Khalil Ghanem: Conceptualization, Funding acquisition, Methodology, Project administration, Writing - review and editing; Rebecca Brotman: Conceptualization, Methodology, Data curation, Writing - original draft, review and editing. All authors read and approved the final version of the manuscript. ST, PG, RB and KG verified the underlying data.

## Data sharing statement

Sequencing data are accessible using the NCBI Sequence Read Archive (SRA) BioProject accession PRJNA610195. A subset of the de-identified metadata variables used in this study (race/ethnicity, age, and hormonal contraceptive use) can be accessed using the NCBI Database of Genotypes and Phenotypes (dbGaP) under accession number phs002169.v1.p1. The remaining survey variables can be accessed directly from the Principal Investigator (Ghanem kghanem@jhmi.edu). Data release queries can also be referred to the Johns Hopkins Medicine Institutional Review Board (jhmeirb@jhmi.edu).

## Declaration of interests

J.R. is co-founder of LUCA Biologics, a biotechnology company focusing on translating microbiome research into live biotherapeutics drugs for women's health. ST has been a consultant for Biofire Diagnostics, Roche Molecular Diagnostics and Luca Biologics, receives royalties from UPTODATE and has received speaker honoraria from Roche Molecular Diagnostics and Medscape. AEB receives university-mediated research funding from 10.13039/100004334Merck and Scope/CHEMO. The remaining authors have not made any declarations.

## Appendix A Supplementary data

MOP_version_brief for publication release 9_27_22

HCL Captions for supplementary material

HCL Supplemental Tables and Methods

HCL Enrollment Forms
