# Supplementary material for: Lactobacillus-dominance and rapid stabilization of vaginal microbiota in combined oral contraceptive pill users examined through a longitudinal cohort study with frequent vaginal sampling over two years
Source: eBioMedicine. 2022 Dec 16;87:104407. doi: 10.1016/j.ebiom.2022.104407 (PMC9792759; doi:10.1016/j.ebiom.2022.104407)
Supplement: MOP_version_brief for publication release 9_27_22 [file mmc1.docx]

**MANUAL OF PROCEDURES**

**Hormonal Contraception Longitudinal Study**

**Version 10 – February 2013 –Abbreviated for Publication Release**

**Dr. Khalil Ghanem**

**Principal Investigator**

### **ACRONYMS**

BSPH Bloomberg School of Public Health

BV Bayview

CT Chlamydia trachomatis

ENRL Enrollment

FACS **Fluorescence-activated cell sorting**

F/U Follow-up

HC Hormonal contraception

ICTR-CRU Institute for Clinical and Translational Research Clinical Research Unit

ID Infectious Diseases

IGS Institute for Genomes Sciences

INT Interval

JHBMC Johns Hopkins Bayview Medical Center

JHBSPH Johns Hopkins Bloomberg School of Public Health

JHMI Johns Hopkins Medical Institutions

JHSON Johns Hopkins School of Nursing

JHSOM Johns Hopkins School of Medicine

JHU Johns Hopkins University

JHWM Johns Hopkins at White Marsh

GC Neisseria gonorrhoeae

GSS Green Spring Station

Myco Mycoplasma genitalium

OB/GYN Obstetrics & Gynecology

OCP Oral contraceptive pill

PBMC Peripheral blood mononuclear cell (i.e. lymphocyte, monocyte, natural killer cell)

PCP Primary Care Provider

PI Principal Investigator

RC Research Clinician

RPMI Roswell Park Memorial Institute

SC Study Coordinator

SCRNG Screening

SOM School of Medicine

STD Sexually-transmitted Disease

ST Study Team

Trich Trichomonas vaginalis

UMD University of Maryland

WM White Marsh

**APPENDIX**

Study Scheme

Recruitment Letter, Brochure & Flyer

Eligibility Form

Short & Long Informed Consent Form

Demographics Questionnaire

Enrollment General Health & Care Questionnaire

Diet Questionnaire

Enrollment Oral Health & Activity Questionnaire

Enrollment Reproductive Health & Activity Questionnaire

Enrollment Clinical Exam & Evaluation Form

Quick-Method Protocol

Interval Daily Diaries & Instructions

Follow-up General Health & Care Questionnaire

Follow-up Oral Health & Activity Questionnaire

Follow-up Reproductive Health & Activity Questionnaire

Follow-up Clinical Exam & Evaluation Form

Status Event Form

Adverse Event Form

**RECRUITMENT**

Recruitment will primarily occur through the BV, WM and GSS OB/GYN Clinics. The ST will obtain a list of all women ages 16-35 scheduled for annual or HC initiation appointments at these clinics one-to-two weeks prior to their appointments. We will write to these women, inviting them to participate in the study (See “Appendix – Recruitment Letter & Brochure”).

Interested women may or may not contact the ST for screening. As such, the ST will visit these clinics and approach women who received recruitment materials in their exam rooms to see if they are interested or not. Regarding women who did not receive recruitment materials, we will wait for their providers to ask them if they are willing to speak with us about the study before approaching them.

Additional recruitment avenues include advertising at the JHBSPH, JHSON, JHSOM and JHU Homewood Campus (See “Appendix – Recruitment Flyer”). Any women enrolled from such areas must have a PCP and/or OB/GYN provider either within JHMI or elsewhere.

**SCREENING**

The ST will receive all interested women at the BV, WM or GSS OB/GYN Clinics or the ICTR-CRU and immediately assign each woman a screening number (See “Screening Numbering System”). They will then determine in which group each woman would participate. Women who are starting or already using HC would participate in the experimental group, while women who are neither starting nor already using HC would participate in the control group. Experimental and control group women will be age-matched.

**EXCLUSION CRITERIA**

However, regardless of in which group each woman would participate, she cannot participate if she:

- had a hysterectomy or intrauterine device implantation
- has a condition altering immune responses (i.e. diabetes mellitus, HIV, etc.)
- has a condition altering sex hormone cycles (i.e. polycystic ovarian syndrome, premature ovarian failure, etc.)
- has a condition contraindicating HC use (i.e. history of thromboembolism, estrogen-dependent tumor, liver disease, pregnancy at HC initiation and/or undiagnosed abnormal uterine bleeding)
- cannot speak English

Please note: before women already using HC can be enrolled, they must indicate that they might stop using it within the next two years.

(See “Appendix – Eligibility Form”)

**SCREENING NUMBERING SYSTEM**

The ST will assign each interested woman a four-digit screening number. The member of the ST conducting the screening will use the first letter of his or her last name as the first digit. The remaining three digits will start at 001 and continue in sequence. As such, the 53^rd^ woman screened by study coordinator last name Jones would have the following screening number: J053.

**ENROLLMENT**

The ST will receive all screened women at the BV, WM, or GSS OB/GYN Clinics or the ICTR-CRU. They will discuss the study responsibilities, risks and rewards with each woman and obtain her informed consent (See “Patient Responsibilities,” “Patient Risks,” “Patient Rewards,” “Informed Consent” and “Appendix: Informed Consent Form”). Once screened women agree to participate in the study, the ST will immediately assign each woman an enrollment number.

**QUESTIONNAIRES**

The ST will then administer questionnaires to each patient, assessing her social and medical history generally and reproductive history specifically (See “Appendix: Demographics Questionnaire,” “Appendix: Diet Questionnaire,” “Appendix: Enrollment General Health & Care Questionnaire,” “Appendix: Enrollment Oral Health & Activity Questionnaire” and “Appendix: Enrollment Reproductive Health & Activity Questionnaire”). Then a clinician will examine and evaluate each patient and obtain specimens (See “Appendix: Enrollment Clinical Exam & Evaluation Form”).

NOTE: The BV, WM, or GSS OB/GYN Clinic staff may interrupt this process at any time. In such an event, each patient will simply complete the questionnaires when she is next able to do so.

**SPECIMENS**

The SC, a member of the BV, WM, or GSS Phlebotomy, or ICTR-CRU Nursing staff will collect 30 ml blood, which the ST will receive. That blood will then be transported to the PI Laboratory at the earliest opportunity. (See “BV and JH Requisitions”).

Meanwhile, the patient will self-collect 2 ml saliva and some urine. The SC will mix the 2 ml saliva and 2 ml RNALater and pipette the liquid equally into 4 aliquots. At least 1 ml urine will be sent to the BV or JH Clinical Pathology Laboratories for culture testing. The rest will be pipetted equally into 4 aliquots.

In addition, the clinician will collect the following specimens in order: 1 dacron vaginal swab (D1), 2 Copan vaginal swabs (C1 & C2), 1 Starplex vaginal swab (S1 & S2), 1 GenProbe vaginal swab (G1), 2 dacron ectocervical and endocervical swabs, 1 endocervical brush (R1) and 1 Copan rectal swab, if the patient does not object.

A vaginal smear (SME) will be obtained with the double-headed Starplex vaginal swab. S2 will be first used for a wet prep, and then saved. A Pap smear (PAP) will be obtained with R1.

The SC will mix C1 in a tube containing 1 ml Liquid Amies and C2 in a tube containing 1 ml RNALater. In addition, she will mix D1 in 5 ml PBS and pipette the liquid equally into 5 aliquots (DA). The same will be done with the dacron ectocervical and endocervical swabs (See “Appendix – Specimen Materials”).

All specimens will be appropriately labeled (See “Specimen Labeling”), kept on ice at the clinic and then transported to the PI Laboratory at the earliest opportunity.

All patients initiating HC will receive real-time pregnancy, STD and HIV testing according to the protocols of the BV, WM, and GSS OB/GYN Clinics. As such, these patients will assume the expense of this testing. However, all patients not initiating HC will receive this testing at our expense if necessary. In such an event, pregnancy testing will be performed in the BV, WM, or GSS OB/GYN Clinics, STD testing will be performed through the JHU STD Laboratory and HIV testing will be performed through the BV or JH Clinical Pathology Laboratories.

The SC will either direct experimental group patients to the clinician for HC initiation or schedule them for a HC initiation appointment. She will schedule control group patients for their next F/U visit and provide them with instructions and materials for the next visit interval (See “Appendix – Visit Interval Daily Diaries & Instructions”). Control group patients will leave the clinic with a specimen collection kit and a $50 check for completing the visit.

**PATIENT RESPONSIBILITIES**

Patients are expected to report to the BV, WM, or GSS OB/GYN Clinics or the ICTR-CRU for their SCRNG/ENRL visit and the ICTR-CRU for each of their seven F/U visits. During these visits, they are expected to cooperate with the ST by answering questions and providing specimens (See “Enrollment” and “Follow-ups”). During the intervals between these visits, patients are expected to observe and indicate vaginal health, activity and pH values, as well as provide vaginal specimens (See “Intervals”).

**PATIENT RISKS**

**CLINICAL**

Risks from venipuncture include discomfort, irritation, bruising and, rarely, thrombophlebitis and infection. Risks from pelvic examination include discomfort and mild bleeding. Because of the number of procedures required, their length might be increased by several minutes, which might increase their associated risks somewhat, but should not do so significantly. In general, these procedures are routinely performed and quite safe.

**TECHNICAL**

All patient information obtained will be kept in respective patient files at respective clinics. Additional information required for the study will be kept in the PI’s office under lock and key. All data will be transferred to a digital encrypted and password-protected database, which the PI can access. In that database, enrollment numbers will be used as unique patient identification codes. The link between these enrollment numbers and patient identifications will be kept in the PI’s office under lock and key. These precautions will be taken to minimize the risk patient information mishandling.

**PATIENT REWARDS**

For assuming the responsibilities and risks associated with the study, patients will receive $50 for the first SCRNG/ENRL visit and each of the seven follow-up visits. They will be reimbursed for any transportation costs incurred by providing parking passes and bus tokens. In addition, if they complete 80% or more of their visits and specimen collections, they will receive $100 as a bonus.

**INFORMED CONSENT**

Informed consent is a process by which individuals voluntarily express their willingness to participate in a study, after having been informed of all its aspects. In order for individuals to properly provide their informed consent, four things must occur: 1) they must receive information about all aspects of the study and 2) understand it. They also must then 3) voluntarily agree to participate in the study and 4) their agreement must be documented. Any woman who is unable to provide her informed consent cannot participate in the study.

In order to ensure these four things occur, the ST will privately discuss the study responsibilities, risks and rewards with each woman and then ask her if she wants to participate. The ST will assure the patient that, should she refuse to participate in the study or choose to participate and withdraw at any time, she will continue to receive excellent care at the BV, WM, or GSS OB/GYN Clinics and other JHMI.

Should the patient choose to participate in the study, a copy of her informed consent form will be placed and maintained in the PI’s office under lock and key for a period of at least 7 years, in compliance with NIH research guidelines and regulations. If space becomes an issue in the PI’s office, he will scan the informed consent forms and store the originals in a secure, off-site location.

NOTE: If there is limited time before interested women see their providers, the ST will obtain the short informed consent form, instead of the long one. This form will allow the collection of genital specimens only from women who sign it. At the earliest opportunity after these women are finished their non-study-related visits, the ST will go through the long informed consent form. In other words, the short informed consent form is *not* a substitute for the long one.

Genital specimens will be immediately destroyed if women, after having gone through the long informed consent form, no longer want to participate in the study. If, on the other hand, they still do, enrollment will continue as planned, with the exception of the physical and gynecological exam and genital specimen collection, as these have already been completed.

If there is enough time to go through the long informed consent form before the interested women see their providers, the short informed consent form will not be used at all.

**HC INITIATION**

The clinician and/or the BV, WM, or GSS OB/GYN Clinic providers will examine and evaluate each patient initiating HC. They will review her medications, check her blood pressure and administer breast and pelvic examinations as well as routine, real-time pregnancy, STD and HIV testing. They will then determine the appropriate HC using the “Quick-Method Protocol” and counsel her about its proper use and associated risks (See “Appendix – Quick-Method Protocol”). They will encourage safe-sex practices by providing counseling and condoms and otherwise initiate HC according to the protocols of the JHBMC OB/GYN Clinic.

Once each patient initiates HC, the ST will schedule her next F/U visit and provide her with instructions and materials for the next visit interval (See “Appendix – Visit Interval Daily Diaries & Specimen Collection Instructions”). Each patient will leave the clinic with a specimen collection kit and a $50 check for completing the visit.

If a patient does not initiate HC on the day of enrollment, the SC will contact her on the planned day of HC initiation to schedule her next F/U visit.

**INTERVALS**

The SC will contact each patient two weeks prior to each of her F/U visits, reminding her to complete the daily diaries and collect 18 vaginal swabs, 6 vaginal smears and 6 vaginal pH measurements over the next two weeks. She will also remind each patient of her upcoming appointment a day prior to it.

**FOLLOW-UPS 1-7**

Each patient will report to the JHBMC ICTR-CRU for her F/U visits. There will be 7 F/U visits total:

- 2 weeks – days 10-18
- 4 weeks – days 23-37
- 3 months – days 76-104
- 6 months – days 150-210
- 12 months – days 330-390
- 18 months – days 510-570
- 24 months – days 690-750

NOTE: If a patient already has an appointment scheduled at the BV, WM, or GSS OB/GYN Clinics within one of her F/U time frames, the SC will report to that clinic, instead of the JHBMC ICTR-CRU, for that F/U.

**QUESTIONNAIRES**

During each F/U visit, the ST will obtain an interval history from each patient, establishing her HC use among other things (See “Appendix – Follow-up General Health & Care Questionnaire,” “Diet Questionnaire,” “Follow-up Oral Health & Activity Questionnaire,” “Appendix – Follow-up Reproductive Health & Activity Questionnaire” and “Appendix – HC Continuation Form”).

The patient need only complete the “Diet Questionnaire” and/or the “Follow-up Oral Health & Activity Questionnaire” if her diet and/or oral health and activity changed significantly since her last visit. Then the clinician will examine and evaluate each patient and obtain specimens (See “Appendix: Follow-up Clinical Exam & Evaluation Form”).

**SPECIMENS**

An ICTR-CRU nurse or the SC will collect 30 ml blood, which the ST will receive. That blood will then be transported to the PI Laboratory at the earliest opportunity.

Meanwhile, the patient will self-collect 2 ml saliva and some urine. The SC will mix the 2 ml saliva and 2 ml RNALater and pipette the liquid equally into 4-6 aliquots. 2 ml urine will be sent to the JHBMC Clinical Pathology Laboratories for culture testing. The rest will be pipetted equally into 4-6 aliquots. A urinalysis test will be performed in one of the aliquots.

In addition, the clinician will collect the following specimens in order: 1 dacron vaginal swab (D1), 2 Copan vaginal swabs (C1 & C2), 1 Starplex vaginal swab (S1 & S2), 1 GenProbe vaginal swab (G1), 2 dacron ectocervical and endocervical swabs, 1 endocervical brush (R1) and 1 Copan rectal swab, if the patient does not object.

A vaginal smear (SME) will be obtained with the double-headed Starplex vaginal swab. S2 will be used for a wet prep as well, and then saved. A Pap smear (PAP) will be obtained with R1.

The SC will mix C1 in a tube containing 1 ml Liquid Amies and C2 in a tube containing 1 ml RNALater. In addition, she will mix D1 in 5 ml PBS and pipette the liquid equally into five aliquots (DA). The same will be done with the dacron ectocervical and endocervical swabs (See “Appendix – Specimen Materials”).

All specimens will be appropriately labeled (See “Specimen Labeling”), kept on ice at the clinic, and then transported to the PI Laboratory daily at the earliest opportunity.

The clinician will not repeat pregnancy, STD and/or HIV testing unless the patient is symptomatic, requests it or the visit interval history warrants it. All testing will be performed at our expense.

The SC will schedule each patient for her next F/U visit and provide her with instructions and materials for the next visit interval (See “Appendix – Visit Interval Daily Diaries” and “Appendix – Visit Interval Specimen Collection Instructions”). Each patient will leave the clinic with a specimen collection kit and a $50 check for completing the visit.

**SPECIMEN MATERIALS**

Each patient will provide the following specimens at the following times (See “Figure 1”):

**Figure 1. Specimen collection scheme.** This outlines what specimens will be collected or received from each patient at or during each visit or visit interval.

| **ENRL** |  | **F/U 1** | | **F/U 2** | | **F/U 3** | | **F/U 4** | | **F/U 5** | | **F/U 6** | |  | **F/U 7** |
| --- | --- | --- | --- | --- | --- | --- | --- | --- | --- | --- | --- | --- | --- | --- | --- |
|  | **INT 1** | | **INT 2** | | **INT 3** | | **INT 4** | | **INT 5** | | **INT 6** | | **INT 7** | |  |
|  | **2 wks** | | **2 wks** | | **2 mos** | | **3 mos** | | **6 mos** | | **6 mos** | | **6 mos** | |  |
|  | 18 v.swabs | | 18 v.swabs | | 18 v.swabs | | 18 v.swabs | | 18 v.swabs | | 18 v.swabs | | 18 v.swabs | |  |
|  | 6 v.smears | | 6 v.smears | | 6 v.smears | | 6 v.smears | | 6 v.smears | | 6 v.smears | | 6 v.smears | |  |
| **0 days** |  | **2 wks** | | **4 wks** | | **3 mos** | | **6 mos** | | **12 mos** | | **18 mos** | |  | **24 mos** |
| 30 ml blood |  | 30 ml blood | | 30 ml blood | | 30 ml blood | | 30 ml blood | | 30 ml blood | | 30 ml blood | |  | 30 ml blood |
| 5 ml saliva |  | 5 ml saliva | | 5 ml saliva | | 5 ml saliva | | 5 ml saliva | | 5 ml saliva | | 5 ml saliva | |  | 5 ml saliva |
| urine |  | urine | | urine | | urine | | urine | | urine | | urine | |  | urine |
| 5 vag.swabs |  | 5 vag.swabs | | 5 vag.swabs | | 5 vag.swabs | | 5 vag.swabs | | 5 vag.swabs | | 5 vag.swabs | |  | 5 vag.swabs |
| 1 ecto.swab |  | 1 ecto.swab | | 1 ecto.swab | | 1 ecto.swab | | 1 ecto.swab | | 1 ecto.swab | | 1 ecto.swab | |  | 1 ecto.swab |
| 1 endo.swab |  | 1 endo.swab | | 1 endo.swab | | 1 endo.swab | | 1 endo.swab | | 1 endo.swab | | 1 endo.swab | |  | 1 endo.swab |
| 1 endo.brush |  | 1 endo.brush | | 1 endo.brush | | 1 endo.brush | | 1 endo.brush | | 1 endo.brush | | 1 endo.brush | |  | 1 endo.brush |
| 1 rect.swab? |  | 1 rect.swab? | | 1 rect.swab? | | 1 rect.swab? | | 1 rect.swab? | | 1 rect.swab? | | 1 rect.swab? | |  | 1 rect.swab? |
| 1 vag.smear |  | 1 vag.smear | | 1 vag.smear | | 1 vag.smear | | 1 vag.smear | | 1 vag.smear | | 1 vag.smear | |  | 1 vag.smear |
| 1 Pap smear |  | 1 Pap smear | | 1 Pap smear | | 1 Pap smear | | 1 Pap smear | | 1 Pap smear | | 1 Pap smear | |  | 1 Pap smear |

The following materials will be used to collect these specimens (See “Table 1”):

**Table 1. Specimen collection materials.** This outlines what materials will be used to collect and store specimens from each patient at or during each visit or visit interval.

| **ENRL & F/U 1-7** | | **INT 1-7** | |
| --- | --- | --- | --- |
| **Specimen** | **Materials** | **Specimen** | **Materials** |
| BLO | (1) 7-ml red-topped tube | VAG | (12) Copan swab |
|  | (4) 2-ml red-topped aliquot (XR) |  | (6) Copan tube with 1 ml LQ Amies (C1) |
|  | (2) 4.5-ml light blue-topped tube |  | (6) 4.5-ml tube with 1 ml RNALater (C2) |
|  | (2) 2-ml clear-topped aliquot (XB) |  | (6) Starplex double-headed swab & tube (S1) |
|  | (2) 5-ml gold-topped tube |  | (6) 5-ml clear-topped tube (S2) |
|  | (4) 2-ml yellow-topped aliquot (XG) | SME | (6) slide |
|  | (1) 4-ml lavender-topped tube |  | (6) slide case |
|  | (2) 2-ml X-topped aliquot (XL) | N/A | (6) pH strip |
| SAL | (1) 15-ml tube with 2 ml RNALater |  |  |
|  | (4) 2-ml white-topped aliquot (XA) |  |  |
| URI | (1) clean-catch urine collection kit |  |  |
|  | (4) 5-ml yellow-topped aliquot (XA) |  |  |
| VAG | (1) dacron swab |  |  |
|  | (1) 5-ml clear-topped tube with 5 ml PBS (D1) |  |  |
|  | (5) 2-ml clear-topped aliquot (DA) |  |  |
| VAG | (2) Copan swab |  |  |
|  | (1) Copan tube with 1 ml LQ Amies (C1) |  |  |
|  | (1) 4.5-ml tube with 1 ml RNALater (C2) |  |  |
|  | (1) Starplex double-headed swab & tube (S1) |  |  |
|  | (1) 5-ml clear-topped tube with 1 ml RNALater (S2) |  |  |
|  | (1) GenProbe swab & tube (G1) |  |  |
| ECT | (1) dacron swab |  |  |
|  | (1) 5-ml blue-topped tube with 5 ml PBS (D1) |  |  |
|  | (5) 2-ml blue-topped aliquot (DA) |  |  |
| END | (1) dacron swab |  |  |
|  | (1) 5-ml green-topped tube with 5 ml PBS (D1) |  |  |
|  | (5) 2-ml green-topped aliquot (DA) |  |  |
| BRU | (1) Rovers brush |  |  |
|  | (1) 5-ml green-topped tube with 2 ml RPMI (R1) |  |  |
|  | (2) 2-ml green-topped aliquot (RA) |  |  |
| REC | (1) Copan swab and tube with 1 ml LQ Amies (C1) |  |  |
| SME | (1) slide |  |  |
|  | (1) slide case |  |  |
| PAP | (1) slide |  |  |
|  | (1) slide case |  |  |

(See “Specimen Labeling”)

**AT THE PI LABORATORY**

The 30 ml blood collected during enrollment and F/U visits will be received at the PI Laboratory. The 7-ml red-topped tube will be stored in 4 2-ml red-topped aliquots. The 2 4.5-ml light blue-topped tubes should yield less than 1 ml of PBMCs, the volume of which will be brought up to 2 ml with RPMI medium. It will then be stored in 2 2-ml clear-topped aliquots. The 2 5-ml gold-topped tubes should yield approximately 4 ml of serum, which will be stored in 4 2-ml yellow-topped aliquots. The 4-ml lavender-topped tube will be stored as is for FACS analysis.

The saliva, urine and Copan rectal swabs collected during enrollment and F/U visits will be received at the PI Laboratory and frozen at -80^o^C for future use.

The vaginal smears collected during enrollment visits, F/U visits and visit intervals will be received at the PI Laboratory at stored at room temperature for Gram staining and Nugent scoring there.

The endocervical brushes collected during enrollment and F/U visits will be received at the PI Laboratory and frozen at -80^o^C for receptor expression testing there. The accompanying Pap smears will likewise be received at the PI Laboratory at stored at room temperature for analysis there.

**STATUS EVENTS**

Approximately 30% of patients are expected to withdraw from the study. However, we expect the majority of patients will successfully conclude the study. That is, they will complete at least 80% of all their patient responsibilities. Despite all this, the following events may occur before a patient concludes the study:

**CROSSOVER**

Approximately 35% of experimental group patients are expected to discontinue their HC regimen. When such a patient does so, the SC and clinician will initiate and continue the F/U visit sequence until the patient has participated in the study for two years.

Approximately 15% of control group patients are expected to initiate a HC regimen. When a control group patient does so, the SC and clinician will initiate and continue the F/U visit sequence until the patient has participated in the study for two years.

**PREGNANCY**

Approximately 3-10% of experimental group patients and 85% of control group patients are expected to become pregnant. When such a patient does so, we will continue the F/U visit sequence until the patient has concluded her two-year study participation, if the patient wishes to continue, that is. If she does, we will proceed as per usual, but we will not collect any cervical specimens. If she does not, on the other hand, she will be discontinued.

(See “Appendix – Status Event Form”)

**ADVERSE EVENTS**

All adverse events will be documented (See “Appendix – Adverse Event Form”). That being said, however, it is unlikely that there will be any events which are truly adverse.
