# Supplementary material for: Lactobacillus-dominance and rapid stabilization of vaginal microbiota in combined oral contraceptive pill users examined through a longitudinal cohort study with frequent vaginal sampling over two years
Source: eBioMedicine. 2022 Dec 16;87:104407. doi: 10.1016/j.ebiom.2022.104407 (PMC9792759; doi:10.1016/j.ebiom.2022.104407)
Supplement: HCL Captions for supplementary material [file mmc2.docx]

**Titles and Captions for Supplementary Material**

**Supplementary Figures**

**Supplementary Figure 1. Hormonal contraception longitudinal study design: vaginal sampling**

**Supplementary Figure 2. Community state types as determined by hierarchical clustering of vaginal microbiota samples.**

Heatmap of relative percent abundance of bacterial taxa found in the vaginal microbiota of 3251 samples from 95 study participants. Vaginal microbiota are grouped into community state types (CSTs) based on bacterial composition and relative abundance. CST I is dominated by *L. crispatus*, CST II by *L. gasseri*, CST III by *L. iners*, CST V by *L. jensenii*, and CST IV is low in *Lactobacillus* spp. and dominated by diverse anaerobes. HC=Hormonal contraceptive, Non-users=participants not on HC for the duration of the study.

**Supplementary Figure 3. Vaginal community state type profiles over time: hormonal contraception users and non-users**.

Vaginal community state types (CSTs) over time in 3251 samples from 95 study participants stratified by hormonal contraception (HC) use, i.e., continuous and intermittent HC users and non-users (never on hormonal contraception during the study). CST I is dominated by L. *crispatus*, CST II by L. *gasseri*, CST III by L. *iners*, CST V by L. *jensenii*, and CST IV is low in *Lactobacillus* spp. and dominated by diverse anaerobes.

**Supplementary Figure 4. Profiles of hormonal contraception usage for the 9 participants who stopped or switched hormonal contraception types.**

Diagram showing hormonal contraception (HC) type over time in the 9 participants who stopped or switched HC type. X-axis shows days of usage and y-axis shows participants.

**Supplementary Figure 5. Vaginal microbiota stability stratified on White participants only: box-percentage plot of Jensen-Shannon indices in samples from COC users versus non-users.**

Amongst White participants, combined estrogen and progestin oral contraceptive pill (COC) users (N=1089 samples from 35 White participants) had increased vaginal microbiota stability as compared to non-users (N=577 samples from 15 White participants). Median Jensen-Shannon indices in COC users: 0.25 versus 0.12 in non-users, difference between COC users and non-users: -0.13, CrI: (-0.17, -0.09).

**Supplementary Figure 6. Vaginal microbiota stability stratified on African American participants only: box-percentage plot of Jensen-Shannon indices in samples from COC users versus non-users.**

Amongst African American participants, combined estrogen and progestin oral contraceptive pill (COC) users (N=202 samples from 7 African American participants) had similar stability as compared to non-users (N=479 samples from 14 African American participants). Median Jensen-Shannon indices in COC users: 0.15 versus 0.15 in non-users, difference: 0; CrI: (-0.08, 0.07).

**Supplementary Figure 7. Vaginal microbiota stability over two years: box-percentage plot of Jensen-Shannon indices in samples from HC users versus non-users.**

Hormonal contraception (HC) users (N=2077 samples from 63 participants) had increased stability as compared to non-users (N=1174 samples from 32 participants). Median Jensen-Shannon indices in HC users: 0.22 versus 0.14 in non-users, difference: -0.08, CrI: (-0.11, -0.038).

**Supplementary Figure 8. Vaginal microbiota stability stratified on White participants only: box-percentage plot of Jensen-Shannon indices in samples from HC users versus non-users.**

Among White participants, hormonal contraception (HC) users (N=1375 samples from 40 White participants) had increased stability as compared to non-users (N=577 samples from 15 White participants). Median Jensen-Shannon indices in HC users: 0.25 versus 0.12 in non-users, difference: -0.13, CrI: (-0.17, -0.09).

**Supplementary Figure 9. Vaginal microbiota stability stratified on African American participants only: box-percentage plot of Jensen-Shannon indices in samples from HC users versus non-users.**

Among African American participants, hormonal contraception (HC) users (N=500 samples from 16 African American participants) had increased stability as compared to non-users (N=479 samples from 14 African American participants). Median Jensen-Shannon indices in HC users: 0.15 versus 0.19 in non-users, difference: 0.03, CrI: (-0.04, 0.12).

**Supplementary Figure 10. Increased stability of vaginal microbiota over time in COC users versus non-users, based on CST evenness.**

This plot shows the dependence between probability of combined oral estrogen and progestin contraceptive pill (COC) use and community state type (CST) evenness (i.e. inverse of stability) utilizing samples from COC users and non-users. Red line=expected proportion, Blue line=estimated proportion of COC users as a function of evenness. More stable microbiota based on CST evenness over time were found in participants on COCs as compared to non-users. The log odds ratio for the COC users (utilizing ordinary logistic regression) is -4.7, CI: (-7.6,-2.1), p=<0.01.

**Supplementary Figure 11.** **Increased stability of vaginal microbiota over time in HC users versus non-users, based on CST evenness.**

This plot shows the dependence between probability of hormonal contraception (HC) use and community state type (CST) evenness utilizing samples from HC users and non-users. Red line=expected proportion, Blue line=estimated proportion of HC users as a function of evenness. More stable microbiota based on CST evenness over time were found in participants on HC as compared to non-users not on HC. The log odds ratio for the HC users (utilizing ordinary logistic regression) is -3.9, CI: (-6.7,-1.48), p<0.01.

**Supplementary Figure 12.** **Increased stability of vaginal microbiota over time in HC users versus non-users, based on CST evenness, after removal of samples taken at the time of menses.**

This plot shows the dependence between probability of hormonal contraception (HC) use and community state type (CST) evenness utilizing samples from HC users and non-users, removing samples taken at the time of menses. Red line=expected proportion, Blue line=estimated proportion of HC users as a function of evenness. More stable communities based on CST evenness over time were found in participants on HC as compared to non-users not on HC. The log odds ratio for the HC users (utilizing ordinary logistic regression) is -3.1, CI: (-5.7,-0.9) p value<0.01.

**Supplementary Figure 13. Increased stability of vaginal microbiota over time in White HC users versus non-users (Panel a, left) and similar stability of vaginal microbiota over time in African American HC users over time (Panel b, right), based on CST evenness.**

These plots show the dependence between probability of hormonal contraception (HC) use and community state type (CST) evenness in HC users and non-users in White participants (Panel a, left) and African American participants (Panel b, right). Red line=expected proportion, Blue line=estimated proportion of HC users as a function of evenness. More stable vaginal microbiota based on CST evenness over time were found in White participants on HC as compared to non-users not on HC, however there was no statistically significant difference in African American users. The log odds ratio for White HC users (utilizing ordinary logistic regression) is -5.04, CI: (-8.98,-1.4), p<0.01, and for African American HC users is -1.42, CI: (-5.5, 2.8), p=0.49.

**Supplementary Figure 14. Timeframe after HC initiation in which vaginal microbiota stability increases.**

This plot shows the dependence between the mean of log ratios of the stability index and the length of time from the initiation of hormonal contraception (HC) in 2454 samples from 25 participants. The stability of the vaginal microbiota in the first dense sampling interval after initiation of HC is significantly higher than during initial interval I on average after 3 weeks from the initiation of HC. Four weeks after initiation, the stability of the vaginal microbiota remains constant.

**Supplementary Figure 15. Timeframe after HC initiation in which vaginal microbiota stability increases in White (Panel a, left) and African American (Panel b, right) participants**.

This plot shows the dependence between the mean of log ratios of the stability index and the length of time interval I, from the initiation of hormonal contraception (HC) in White participants (Panel a, left) and African American participants (Panel b, right). The stability of the vaginal microbiota in the first dense sampling interval after initiation of HC is significantly higher than during initial interval I on average 4 weeks from initiation of HCs in White participants and African American participants. Four weeks after initiation, the stability of the vaginal microbiota remains constant.

**Supplementary Tables**

**Supplementary Table 1**. **Proportions of samples from COC users versus non-users in *Lactobacillus*-dominated vs. non-*Lactobacillus*-dominated vaginal community state types.** N=number of samples, Non-Lactobacillus CST=non-*Lactobacillus* dominated community state type, *Lactobacillus* CSTs=*Lactobacillus-*dominated Community State Type, Non-Users=those who never used HC during the entire study, COC=combined estrogen and progestin oral contraceptive pill. Point estimates and the corresponding credible intervals (CrIs) were estimated using Bayesian mixed effects Bernoulli models with subject-wise random intercept (see Methods – Supplemental Materials for details). The point estimates are the log ratios of the estimated proportions of the given CST between COC users and non-users. As such, they are not the log ratios of the sample proportions of the CSTs within COC users and non-users.

**Supplementary Table 2**. **Proportions of samples from COC users versus non-users in each vaginal community state type.** N=number of samples, Non-users=those who never used HC during the entire study, CST=community state type, COC=combined estrogen and progestin oral contraceptive pill. . Point estimates and the corresponding credible intervals (CrIs) were estimated using Bayesian mixed effects Bernoulli models with subject-wise random intercept (see Methods – Supplemental Materials for details). The point estimates are log ratios of the estimated proportions of the given CST between COC users and non-users. As such, they are not the log ratios of the sample proportions of the CSTs within COC users and non-users.

**Supplementary Table 3**. **Proportions of samples from HC users versus non-users in *Lactobacillus*-dominated vs. non-*Lactobacillus*-dominated vaginal Community State Types.** N=number of samples, CST=Community State Type, Non-users=samples from those who never used HC during the entire study, HC=hormonal contraception. Point estimates and the corresponding credible intervals (CrIs) were estimated using Bayesian mixed effects Bernoulli models with subject-wise random intercept (see Methods – Supplemental Materials for details). The point estimates are log ratios of the estimated proportions of the given CST between COC users and non-users. As such, they are not the log ratios of the sample proportions of the CSTs within COC users and non-users.

**Supplementary Table 4. Proportions of samples from HC users versus non-users in each vaginal community state type.** N=number of samples, Non-users=samples from those who never used HC during the entire study, CST=community state type, HC=hormonal contraception. Point estimates and the corresponding credible intervals (CrIs) were estimated using Bayesian mixed effects Bernoulli models with subject-wise random intercept (see Methods – Supplemental Materials for details). The point estimates are log ratios of the estimated proportions of the given CST between COC users and non-users. As such, they are not the log ratios of the sample proportions of the CSTs within COC users and non-users.

**Supplementary Table 5. Baseline (enrollment) characteristics of HC users and non-users including 5 additional patients included in HC initiation analyses.** *Chi-squared tests were utilized except for age variable where t-test was utilized. ******During entire study. ***Including community college. Note that a few patients switched HC types (see Suppl Fig. 3), HC=hormonal contraception, COC=combined oral contraceptive pill, IUD=intrauterine device, Injection=Medroxyprogesterone acetate shot. All implant users used either Nexplanon or Implanon. HS=high school, Grad=graduate
