# Supplementary material for: Lactobacillus-dominance and rapid stabilization of vaginal microbiota in combined oral contraceptive pill users examined through a longitudinal cohort study with frequent vaginal sampling over two years
Source: eBioMedicine. 2022 Dec 16;87:104407. doi: 10.1016/j.ebiom.2022.104407 (PMC9792759; doi:10.1016/j.ebiom.2022.104407)
Supplement: HCL Supplemental Tables and Methods [file mmc3.docx]

**SUPPLEMENTAL TABLES AND METHODS**

**Supplementary Table 1. Proportions of samples from COC users versus non-users in *Lactobacillus*-dominated vs. non-*Lactobacillus*-dominated vaginal community state types (CST).**

|  | Non-*Lactobacillus* CSTs  N (%) | *Lactobacillus* CSTs  N (%) |
| --- | --- | --- |
| **All participants, menses removed** |  |  |
| COC users | 129 (11.2) | 1026 (88.8) |
| Non-users | 233 (22.9) | 783 (77.1) |
| Point estimates and CrIs | -2.63 (-4.82,-0.69) | 0.0722 (0.0075,0.23) |
| **White only** |  |  |
| COC users | 68 (6.2) | 1021 (93.8) |
| Non-users | 93 (16.1) | 484 (83.9) |
| Point estimates and CrIs | -3.29 (-6.28,-0.9) | 0.09 (0.008,0.33) |
| **African American only** |  |  |
| COC users | 73 (36.1) | 129 (63.9) |
| Non-users | 162 (33.8) | 317 (66.2) |
| Point estimates and CrIs | -0.827 (-4.28,2.22) | 0.00175 (-0.97,0.766) |

N=number of samples, Non-Lactobacillus CST=non-*Lactobacillus* dominated community state type, *Lactobacillus* CSTs=*Lactobacillus-*dominated Community State Type, Non-users=samples from those who never used HC during the entire study, COC=combined estrogen and progestin oral contraceptive pill. Point estimates and the corresponding credible intervals (CrIs) were estimated using Bayesian mixed effects Bernoulli models with subject-wise random intercept (see Methods – Supplemental Materials for details). The point estimates are the log ratios of the estimated proportions of the given CST between COC users and non-users. As such, they are not the log ratios of the sample proportions of the CSTs within COC users and non-users.

**Supplementary Table 2. Proportions of samples from COC users versus non-users in each vaginal community state type.**

|  | CST I  N (%) | CST II  N (%) | CST III  N (%) | CST IV  N (%) | CST V  N (%) |
| --- | --- | --- | --- | --- | --- |
| **All participants, menses removed** |  |  |  |  |  |
| COC users | 654 (56.6) | 5 (0.4) | 288 (24.9) | 129 (11.2) | 79 (6.8) |
| Non-users | 397 (39.1) | 51 (5) | 283 (27.9) | 233 (22.9) | 52 (5.1) |
| Point estimates and CrIs | 2.53 (0.57,4.87) | -3.11  (-7.86,1.04) | -0.776  (-2.1,0.51) | -2.62  (-4.75,-0.67) | -0.754  (-4.28,2.8) |
| **White only** |  |  |  |  |  |
| COC users | 696 (63.9) | 5 (0.5) | 285 (26.2) | 68 (6.2) | 35 (3.2) |
| Non-users | 283 (49) | 25 (4.3) | 144 (25.0) | 93 (16.1) | 32 (5.5) |
| Point estimates and CrIs | 1.25  (-0.13,3.35) | -2.18  (-7.79,3) | -0.264  (-2.12,1.75) | -3.26  (-5.94,-0.85) | -1.12  (-4.96,2.25) |
| **African American only** |  |  |  |  |  |
| COC users | 33 (16.3) | 0 (0.0) | 47 (23.3) | 73 (36.1) | 49 (24.3) |
| Non-users | 106 (22.1) | 26 (5.4) | 163 (34.0) | 162 (33.8) | 22 (4.6) |
| Point estimates and CrIs | 3.98 (-3,11.5) | -2.75  (-10.2,4.82) | -1.67  (-4.43,0.57) | -0.84  (-4.26,2.14) | 2.74  (-5.72,11.1) |

N=number of samples, Non-users=samples from those who never used HC during the entire study, CST=community state type, COC=combined estrogen and progestin oral contraceptive pill. Point estimates and the corresponding credible intervals (CrIs) were estimated using Bayesian mixed effects Bernoulli models with subject-wise random intercept (see Methods – Supplemental Materials for details). The point estimates are log ratios of the estimated proportions of the given CST between COC users and non-users. As such, they are not the log ratios of the sample proportions of the CSTs within COC users and non-users.

**Supplementary Table 3. Proportions of samples from HC users versus non-users in *Lactobacillus*-dominated vs. non-*Lactobacillus*-dominated vaginal Community State Types.**

|  | Non-Lactobacillus CSTs  N (%) | Lactobacillus CSTs  N (%) |
| --- | --- | --- |
| **All participants** |  |  |
| HC users | 221 (10.6) | 1856 (89.4) |
| Non-users | 277 (23.6) | 897 (76.4) |
| Point estimates and CrIs | -2.1 (-3.51,-0.73) | 0.096 (0.016,0.28) |
| **All participants, menses removed** |  |  |
| HC users | 192 (11.0) | 1549 (89.0) |
| Non-users | 233 (22.9) | 783 (77.1) |
| Point estimates and CrIs | -1.84 (-3.48,-0.22) | 0.071 (0.0065,0.21) |
| **White only** |  |  |
| HC users | 82 (6.0) | 1293 (94) |
| Non-users | 93 (16.1) | 484 (83.9) |
| Point estimates and CrIs | -3.16 (-5.89,-0.97) | 0.086 (0.0066,0.33) |
| **African American only** |  |  |
| HC users | 126 (25.2) | 374 (74.8) |
| Non-users | 162 (33.8) | 317 (66.2) |
| Point estimates and CrIs | -0.413 (-2.55,1.74) | 0.094 (-0.29,0.61) |

N=number of samples, CST=Community State Type, Non-users=samples from those who never used HC during the entire study, HC=hormonal contraception. Point estimates and the corresponding credible intervals (CrIs) were estimated using Bayesian mixed effects Bernoulli models with subject-wise random intercept (see Methods – Supplemental Materials for details). The point estimates are log ratios of the estimated proportions of the given CST between COC users and non-users. As such, they are not the log ratios of the sample proportions of the CSTs within COC users and non-users.

**Supplementary Table 4. Proportions of samples from HC users versus non-users in each vaginal community state type.**

|  | CST I  N (%) | CST II  N (%) | CST III  N (%) | CST IV  N (%) | CST V  N (%) |
| --- | --- | --- | --- | --- | --- |
| **All participants** |  |  |  |  |  |
| HC users | 1074 (51.7) | 6 (0.3) | 623 (30.0) | 221 (10.6) | 153 (7.4) |
| Non-users | 446 (38) | 52 (4.4) | 344 (29.3) | 277 (23.6) | 55 (4.7) |
| Point estimates and CrIs | 2.3 (0.421,4.49) | -3.11  (-7.02,0.322) | -0.33  (-1.38,0.73) | -2.13  (-3.64,-0.66) | -0.22  (-3.39,2.91) |
| **All participants, menses removed** |  |  |  |  |  |
| HC users | 896 (51.5) | 6 (0.3) | 502 (28.8) | 192 (11.0) | 145 (8.3) |
| Non-users | 397 (39.1) | 51 (5.0) | 283 (27.9) | 233 (22.9) | 52 (5.1) |
| Point estimates and CrIs | 2.07 (0.0677,4.44) | -2.78  (-6.99,0.88) | -0.25  (-1.32,0.8) | -1.89  (-3.72,-0.21) | -0.61  (-4.02,2.67) |
| **White only** |  |  |  |  |  |
| HC users | 835 (60.7) | 5 (0.4) | 406 (29.5) | 82 (6.0) | 47 (3.4) |
| Non-users | 283 (49) | 25 (4.3) | 144 (25) | 93 (16.1) | 32 (5.5) |
| Point estimates and CrIs | 1.13  (-0.34,3.36) | -2.59  (-7.59,2.4) | 0.14  (-1.59,2.13) | -3.12  (-5.75,-0.85) | -1.12  (-4.71,2.29) |
| **African American only** |  |  |  |  |  |
| HC users | 118 (23.6) | 1 (0.2) | 149 (29.8) | 126 (25.2) | 106 (21.2) |
| Non-users | 106 (22.1) | 26 (5.4) | 163 (34.0) | 162 (33.8) | 22 (4.6) |
| Point estimates and CrIs | 2.48 (-3.17,8.22) | -2.25  (-7.54,2.74) | -0.55  (-2.15,0.884) | -0.437  (-2.59,1.71) | 2.8  (-3.61,9.2) |

N=number of samples, Non-users=samples from those who never used HC during the entire study, CST=community state type, HC=hormonal contraception. Point estimates and the corresponding credible intervals (CrIs) were estimated using Bayesian mixed effects Bernoulli models with subject-wise random intercept (see Methods – Supplemental Materials for details). The point estimates are log ratios of the estimated proportions of the given CST between COC users and non-users. As such, they are not the log ratios of the sample proportions of the CSTs within COC users and non-users.

**Supplementary Table 5. Baseline (enrollment) characteristics of HC users and non-users including 5 additional patients included in HC initiation analyses.**

|  | **Overall**  **N=100** | **HC users**  **N=68** | **Non-users**  **N=32** | **P value*** |
| --- | --- | --- | --- | --- |
| **Age (median, interquartile range)** | 25.7 (21.9-29.3) | 24.8 (21.8-28.4) | 26.9 (22.3-31.0) | 0.05 |
| **Race**  White  African American  Other | 57 (57.0)  30 (30.0)  13 (13.0) | 42 (61.8)  16 (23.5)  10 (14.7) | 15 (46.9)  14 (43.8)  3 (9.4) | 0.12 |
| **Number of male sexual partners in last 2 months**  0  1  >=2 | 18 (18.0)  78 (78.0)  4 (4.0) | 12 (17.6)  54 (79.4)  2 (2.9) | 6 (18.8)  24 (75.0)  2 (6.3) | 0.72 |
| **Number of female sexual partners in last 2 months**  0  1  >=2 | 98 (98.0)  2 (2.0)  N/A | 67 (98.5)  1 (1.5)  N/A | 31 (96.9)  1 (3.1)  N/A | 0.58 |
| **Condom use with vaginal sex in last 2 months**  Never  Sometimes  Always  N/A | 39 (39.0)  13 (13.0)  35 (35.0)  13 (13.0) | 29 (42.7)  7 (10.3)  23 (33.8)  9 (13.2) | 10 (31.3)  6 (18.8)  12 (37.5)  4 (12.5) | 0.57 |
| **Types of HC used****  COC  Patch  Ring  Injection  Implant  Hormonal IUD | N/A | 50  2  6  4  5  4 | N/A | N/A |
| **Douching in last 2 months**  >=Once per month  >=Once in 2 months  None | 4 (4.0)  1 (1.0)  95 (95.0) | 2 (2.9)  1 (1.5)  65 (95.6) | 2 (6.3)  0 (0.0)  30 (93.8) | 0.59 |
| **Educational attainment**  HS or some HS  College or some College***  Grad school or some Grad school | 8 (8.0)  62 (62.0)  30 (30.0) | 4 (6.4)  41 (60.3)  23 (33.8) | 4 (12.5)  21 (65.6)  7 (21.9) | 0.31 |

*****Chi-squared tests were utilized except for age variable where t-test was utilized. ******During entire study. ***Including community college. Note that a few patients switched HC types (see Suppl Fig. 3), HC=hormonal contraception, COC=combined oral contraceptive pill, IUD=intrauterine device, Injection=Medroxyprogesterone acetate shot. All implant users used either Nexplanon or Implanon. HS=high school, Grad=graduate

**SUPPLEMENTAL METHODS**

**Additional Information on Participant Recruitment**

All patients who were scheduled to see their providers for HC initiation or discontinuation were identified prior to their clinic visit and an invitation letter was sent to each participant with a phone number to call if they were interested in participating in the study. Controls were selected from the same clinics among patients who were not on HC and were not planning on starting HC. If patients were interested in participating in the study (by contacting us prior to their clinic visit), they were consented during their clinic visit. All study follow-up appointments were scheduled in the research building. Written informed consent was obtained in a private room by trained study staff.

**Data Handling**

All data was entered into Redcap and then exported for analysis.

**Statistical analysis**

**Bayesian logistic regression models: CSTs**

Bayesian logistic regression models for estimating credible intervals of the differences in proportions of CSTs between different groups were estimated using the R package rstan, which is the R interface to the Stan - statistical modeling and high-performance statistical computation platform [S, rS]. The structure of these models was the Bernoulli model with the logit link

y_i∼"bernoulli" (μ_("grou" "p" _"i" )+d_("subjI" "D" _"i" ) ),

where y_i is the value of a binary variable at the i-th sample with "grou" "p" _"i" and subjIDi the group and subject indices of the i-th sample. Thus, the Bernoulli distribution probability of y_i being 1 parameter is the sum of the probability of the i-th group and the random intercept with

d_j∼"normal" (0,σ),

where the priors were σ∼"cauchy" (0,5) and μ∼"normal" (-5,5) with the mean of the μ prior set to -5 making the prior assumption about the proportion of a given CST close to zero.

[S] Stan Development Team (2022). Stan Modeling Language Users Guide and Reference Manual, version 2.30. https://mc-stan.org

[rS] Stan Development Team (2022). “RStan: the R interface to Stan.” R package version 2.21.5, https://mc-stan.org/.

**Stability index:**

The stability index was defined as the median Jensen-Shannon distance from the centroid of the samples for which the index was computed. This stability index was first introduced in Gajer P, Brotman RM, Bai G, Sakamoto J, Schutte UM, Zhong X, et al. Temporal dynamics of the human vaginal microbiota. Sci Transl Med. 2012;4(132):132ra52.
