# Supplementary material for: Lactobacillus-dominance and rapid stabilization of vaginal microbiota in combined oral contraceptive pill users examined through a longitudinal cohort study with frequent vaginal sampling over two years
Source: eBioMedicine. 2022 Dec 16;87:104407. doi: 10.1016/j.ebiom.2022.104407 (PMC9792759; doi:10.1016/j.ebiom.2022.104407)
Supplement: HCL Enrollment Forms [file mmc4.pdf]

HCL Study – Enrollment Visit
Eligibility Form

Date: |____|____| / |____|____| / |____|____| Site #: |____|____|____|
mm dd yy
ENRL #: |__E__|____|____|____| Visit ID: |____|____|____|____| Staff ID: |____|____|

Fill in all with which you agree.

1. O She is 16-35 years of age
2. O She can speak English
3. O She is ***not*** pregnant
4. O She has ***not*** had a hysterectomy
5. O She does ***not*** have an intrauterine device implantation
6. O She does ***not*** have any of the following conditions, which suppress her immune system. (If she ***does***, fill in all that applies.)
   a. O Diabetes
   b. O HIV
   c. O Other, specify______________________
7. O She is ***not*** taking any medications, which suppress her immune system
8. O She does ***not*** have any of the following conditions, which alter her sex hormones. (If she ***does***, fill in all that applies.)
   a. O Polycystic ovarian syndrome
   b. O Premature ovarian failure
   c. O Other, specify______________________
9. O She has ***not*** had or does ***not*** have any of the following conditions, which contraindicate HC use. (If she ***does***, fill in all that applies.)
   a. O Thromboembolism
   b. O Estrogen-dependent tumor
   c. O Liver disease
   d. O Abnormal uterine bleeding
   e. O Other, specify______________________
10. O She is not currently on birth control.
    O She is newly starting birth control.
    O She is already on birth control, and she has indicated that she could discontinue it within the next two years.

If you filled in items 1-10, enroll patient and complete items 11-15.

11. O She signed the informed consent form while being observed by a witness.
12. O The witness signed the informed consent form.
13. O One copy of the signed informed consent form was given to her and another was retained in her study record.
14. Date signed informed consent:
    |____|____| / |____|____| / |____|____|
15. Assigned patient enrollment number:
    |__E__|____|____|____|

Initials of person completing the form: ________________ Date: _____________________

HCL Study – Enrollment Visit
Demographics Questionnaire

Date: |____|____| / |____|____| / |____|____| (mm dd yy) Site #: |____|____|____|

ENRL #: |__E__|____|____|____| Visit ID: |____|____|____|____| Staff ID: |____|____|

1. What is your date of birth? .... |____|____| / |____|____| / |____|____| (mm/dd/yy)

2. How old are you? .... |____|____| yr

Fill in all that applies.

3. Which of the following describes your race or ethnicity?

- O Asian/Pacific Islander
- O Black/African American
- O Hispanic/Latina
- O Native American/ American Indian
- O White/Caucasian
- O Other
- O No answer

If "Other," specify____

4. How much school have you completed?

- O None
- O Some elementary school
- O Elementary school (1st-5th)
- O Some middle school
- O Middle school (6th-8th)
- O Some high school
- O High school (9th-12th)
- O Some community college
- O Community college (2 years)
- O Some college
- O College (4 years)
- O Some graduate school
- O Graduate school
- O No answer

5. During the last year, where did you get money for food, clothing and shelter?

- O Job
- O Own business
- O Significant other
- O Family or friends
- O Alimony payments
- O Welfare payments, such as AFDC or SSI (WIC, Food Stamps, Free School lunches, etc.)
- O Unemployment benefits
- O Other
- O No answer

If "Other," specify____

6. What is your total household income from all sources per month?

- O $500 or less
- O $501-800
- O $801 – 3000
- O $3001 or more
- O No answer

7. What is your marital status?

- O Never married
- O Married
- O Separated
- O Divorced
- O Widowed
- O Other
- O No answer

If "Other," specify____

Initials of person completing the form: ________________ Date: ______________________

HCL Study – Enrollment Visit
General Health & Care Questionnaire

Date: |____|____| / |____|____| / |____|____| (mm dd yy)   Site #: |____|____|____|

ENRL #: |__E__|____|____|____| Visit ID: |____|____|____|____| Staff ID: |____|____|

1. Are you in good general health? ○ Yes (skip to item 2) ○ No

a. If "No", briefly describe______________________________

2. Some people visit a doctor just for a checkup. How long ago was your last checkup?

○ Less than 1 year ago
○ Between 1 and 3 years ago
○ More than 3 years ago
○ I do not visit a doctor just for a checkup.
○ No answer

3. Do you have any allergies? ○ Yes ○ No (skip to item 4)

a. If "Yes", specify______________________________

4. Do you know you have any of the following? (Fill in all that applies.)

○ Diabetes
○ Hypertension
○ Heart disease Specify____________
○ Lung disease Specify____________
○ Liver disease Specify____________
○ Kidney disease Specify____________
○ Gastrointestinal disease Specify____________
○ Skin disease Specify____________
○ Immunological disorder Specify____________
○ Rheumatic disorder Specify____________
○ Other Specify____________
○ None
○ No answer

5. Do you take any vitamins or other supplements? ○ Yes ○ No (skip to item 6)

a. If "Yes", specify______________________________

6. Do you take any prescription medications? ○ Yes ○ No (skip to item 7)

a. If "Yes", please list all your medications here and provide start and end dates for each.

____

____

7. Do you take any over-the-counter medications? ○ Yes ○ No (skip to item 8)

a. If "Yes", please list all your medications here and provide start and end dates for each.

____

____

8. In the last 30 days, have you taken or are you currently taking any antibiotics? ○ Yes ○ No (skip to item 9)

a. If "Yes", please list all your antibiotics here and provide start and end dates for each.

____

9. In the last 30 days, have you taken or are you currently taking any anti-fungals? ○ Yes ○ No (skip to end)

a. If "Yes", please list all your anti-fungals here and provide start and end dates for each.

____

Initials of person completing the form: ____________ Date: ______________

HCL Study – Enrollment Visit
Hormonal Contraception Form

Date: |____|____| / |____|____| / |____|____| Site #: |____|____|____|
mm dd yy
ENRL #: |__E__|____|____|____| Visit ID: |____|____|____|____| Staff ID: |____|____|

If she is not starting or currently on HC, please strike through this form.

1. **What HC are you starting or currently on?**

   ○ Pill, specify ________________________________

   ○ Patch, specify ________________________________

   ○ Ring, specify ________________________________

   ○ Injection, specify ________________________________

   ○ Other, specify ________________________________

2. **If she is starting HC, when will she start it?**....................................|____|____| / |____|____| / |____|____| (mm/dd/yy)

3. **If she is currently on HC, when did she start it?**.............................|____|____| / |____|____| / |____|____| (mm/dd/yy)

(If she remembers the date, write the date. If she only remembers, the month and year, but not the day, write the 1st of the month. If she remembers the year, but not the month or the day, write January 1st of the year.)

For current HC users ONLY:

4. **If you take the pill, have you ever missed any in the last 6 months?** ○ No ○ Yes

   a. **If yes, what percentage of pills, on average, did you miss in the last 6 months?**
   ○ 0-15% ○ 16-30% ○ 31-45% ○ 46-60% ○ 61-85% ○ 86-100%

5. **If you wear the patch, have you ever <u>not</u> worn it when you were supposed to in the last 6 months?** ○ No ○ Yes

   a. **If yes, what percentage of the time, on average, did you <u>not</u> wear it in the last 6 months?**
   ○ 0-15% ○ 16-30% ○ 31-45% ○ 46-60% ○ 61-85% ○ 86-100%

6. **If you wear the ring, have you ever <u>not</u> worn it when you were supposed to in the last 6 months?** ○ No ○ Yes

   a. **If yes, what percentage of the time, on average, did you <u>not</u> wear it in the last 6 months?**
   ○ 0-15% ○ 16-30% ○ 31-45% ○ 46-60% ○ 61-85% ○ 86-100%

7. **If you receive injections, have you ever missed one in the last 6 months?** ○ No ○ Yes

   a. **If yes, how many, on average, have you missed?** ○ 1 ○ 2

8. **Have you experienced any irregular symptoms, such as bleeding in the last 6 months?** ○ No ○ Yes

   Comments: ________________________________

   ____

   ____

Initials of person completing the form: ________________ Date: ____________________

HCL Study – Enrollment Visit
HIV Form

Date: |____|____| / |____|____| / |____|____| Site #: |____|____|____|
mm dd yy
ENRL #: |__E__|____|____|____| Visit ID: |____|____|____|____| Staff ID: |____|____|

If she does not have HIV, please strike through this form.

For questions involving dates: If she remembers the date, write the date. If she only remembers, the month and year, but not the day, write the 1st of the month. If she remembers the year, but not the month or the day, write January 1st of the year.

For questions involving medications: Please provide doses and starting and ending dates for each.

1. **When were you diagnosed with HIV?**..|____|____| / |____|____| / |____|____| (mm/dd/yy)

2. **To the best of your knowledge, how did you get HIV?**
   - O **Homosexual contact**
   - O **Heterosexual contact**
   - O **IV drug use**
   - O **Mother to child**
   - O **Transfusion recipient**
   - O **Other:** ____
   - O **Unknown**
   - O **No answer**

3. **Where do you receive HIV care?** ____
   a. **When was your last HIV-related doctor's visit?**....................|____|____| / |____|____| / |____|____| (mm/dd/yy)

4. **When was your CD4 count last measured?**...................................|____|____| / |____|____| / |____|____| (mm/dd/yy)
   a. **What was it?** |____|____|____| cells/μl O Unknown O No answer
   b. **What was your lowest CD4 count?** |____|____|____| cells/μl O Unknown O No answer

5. **When was your viral load last measured?**.....................................|____|____| / |____|____| / |____|____| (mm/dd/yy)
   a. **What was it?** |____|____|____| copies/ml O Unknown O No answer

6. **Are you on HAART therapy?** O Yes O No O No answer
   a. **If yes, what HAART regimen are you on/what HAART medications do you take?**
   ____
   ____
   ____
   b. **What other HAART regimens have you been on/what HAART medications have you taken in the past six months?**
   ____
   ____
   ____

7. **Have you taken any non-HAART HIV-related medications in the past 6 months?** O Yes O No O No answer
   ____
   ____

Initials of person completing the form: ________________ Date: ______________________

HCL Study – Enrollment Visit
Oral Health & Activity Questionnaire

Date: |____|____| / |____|____| / |____|____| Site #: |____|____|____|
mm dd yy
ENRL #: |__E__|____|____|____| Visit ID: |____|____|____|____| Staff ID: |____|____|

1. **Do you have a dental checkup at least every 2 years?** O Yes O No O No answer

2. **Have you seen a dentist in the last 6 months?** O Yes O No O No answer

3. **In the last 6 months, how often have you been brushing your teeth?**
   - O Twice per day or more
   - O Once per day
   - O At least once per week
   - O At least once per month
   - O Less than once per month
   - O I do not brush my teeth.
   - O No answer

4. **In the last 6 months, how often have you been flossing your teeth?**
   - O Twice per day or more
   - O Once per day
   - O At least once per week
   - O At least once per month
   - O Less than once per month
   - O I do not floss my teeth.
   - O No answer

5. **In the last 6 months, how often has your gum been bleeding when you brushed/flossed your teeth?**
   - O Always
   - O Most of the time
   - O About half of the time
   - O Seldom
   - O Never
   - O I do not brush/floss my teeth.
   - O No answer

6. **In the last year, have you had any toothaches or teeth pulled?** O Yes O No O No answer

7. **In the last 2 months, have you used any of the following? [Specify how many (i.e. 1, 2, 3, etc.) you have used per time frame (i.e. day, wk, mo, etc.]**

O Cigarettes	Specify___________________	O Snuff	Specify____________________
O Cigars	Specify___________________	O Other: _____________	Specify____________________
O Pipe tobacco	Specify___________________	O None (Skip to item 9)	
O Chewing tobacco	Specify___________________	O No answer	

8. **In the last week, have you used any of the following? [Specify how many (i.e. 1, 2, 3, etc.) you have used per time frame (i.e. day, wk, mo, etc.]**

O Cigarettes	Specify___________________	O Snuff	Specify____________________
O Cigars	Specify___________________	O Other: _____________	Specify____________________
O Pipe tobacco	Specify___________________	O None	
O Chewing tobacco	Specify___________________	O No answer	

9. **In the last 2 months, have you used any of the following? (Fill in all that applies.)**
   - O Powdered cocaine
   - O Crack cocaine
   - O Methadone
   - O Methamphetamine
   - O Marijuana
   - O Narcotics, specify________________
   - O Other, specify___________________
   - O None
   - O No answer

10. **In the last week, how often have you drunk beer, wine or other alcohol?**
    - O About every day (5-7 days)
    - O Several times (2-4 days)
    - O Once
    - O Never (Skip to end)
    - O No answer

    a. **On the days you drank, how many drinks did you usually have?**
       O __________ O No answer

Initials of person completing the form: _______________ Date: ____________________

Date: |____|____| / |____|____| / |____|____|

ENRL #: |__E__|____|____|____|

ENRL **REPROD**

HCL Study – Enrollment Visit
Reproductive Health & Activity Questionnaire

For staff only: Site #: |____|____|____| Visit ID: |____|____|____|____| Staff ID: |____|____|

1. **Date of last menstrual period** ....|____|____| / |____|____| / |____|____| (mm/dd/yy)

2. **How long was your last menstrual period?**
   - O 1-2 days
   - O 3-4 days
   - O 5-6 days
   - O More than 6 days
   - O No answer

3. **How heavy was your last menstrual period?** O Light O Moderate O Heavy O No answer

4. **What sanitary protection did you use during your last menstrual period? (Fill in all that applies.)**
   - O Tampons
   - O Sanitary pads
   - O Panty liners
   - O Cloth
   - O Tissue
   - O Other: __________________________
   - O None
   - O No answer

5. **Did you use tampons at times other than your last menstrual period?** O No O Yes O No answer

   a. **For what reason do you use tampons at these times? (Fill in all that applies.)**
   - O Cleanliness
   - O Odor or smell
   - O Vaginal discharge
   - O Blood spotting
   - O Habit
   - O Other: ________________________
   - O No answer

6. **Did you use other sanitary protection at times other than your last menstrual period?** O No O Yes O No answer

   a. **For what reason do you use other sanitary protection at these times? (Fill in all that applies.)**
   - O Cleanliness
   - O Odor or smell
   - O Vaginal discharge
   - O Blood spotting
   - O Habit
   - O Other: ________________________
   - O No answer

7. **In the last 2 months, have you had any of these symptoms? (Fill in what you experienced and how often you experienced it.)**

O Staining of the underwear ➤	O 1 X / 2 mo	O 1-3 X / mo	O 1-3 X / wk	O 4-6 X / wk	O Daily	O No answer
O Vaginal discharge ➤	O 1 X / 2 mo	O 1-3 X / mo	O 1-3 X / wk	O 4-6 X / wk	O Daily	O No answer
O Vaginal odor ➤	O 1 X / 2 mo	O 1-3 X / mo	O 1-3 X / wk	O 4-6 X / wk	O Daily	O No answer
O Vaginal irritation ➤	O 1 X / 2 mo	O 1-3 X / mo	O 1-3 X / wk	O 4-6 X / wk	O Daily	O No answer
O Persistent vaginal itch ➤	O 1 X / 2 mo	O 1-3 X / mo	O 1-3 X / wk	O 4-6 X / wk	O Daily	O No answer
O Pain during urination ➤	O 1 X / 2 mo	O 1-3 X / mo	O 1-3 X / wk	O 4-6 X / wk	O Daily	O No answer
O Abdominal or pelvic pain ➤	O 1 X / 2 mo	O 1-3 X / mo	O 1-3 X / wk	O 4-6 X / wk	O Daily	O No answer
O None						
O No answer						

8. **In the last 2 months, have you had any of these conditions diagnosed by a clinician? (Fill in all that applies.)**
   - O Yeast infection
   - O Bacterial vaginosis
   - O Chlamydia
   - O Trichomonas
   - O Gonorrhea
   - O Genital wart (HPV)
   - O Herpes
   - O Syphilis
   - O Pelvic inflammatory disease
   - O Other: __________________________
   - O None
   - O No answer

9. **In the last 5 weeks, have you self-treated any vaginal conditions with over-the-counter medications such as Gyne-Lotrimin or Monistat?** O No (skip to item 10) O Yes O No answer

   a. **With what over-the-counter medication did you self-treat your vaginal condition?**
   O Lotrimin O Monistat O Other: __________________________ O No answer

10. **You would describe your normal vaginal odor as:**
    - O Fishy
    - O Foul (bad body odor)
    - O Musty
    - O Other: __________________________
    - O I do not normally have an odor.
    - O No answer

11. **Have you ever douched? By douching, we mean flushing out your vagina with some fluid.**
    O No (skip to item 15) O Yes O No answer

12. **In the last 2 months, how frequently have you douched?**
   - O More than once a day
   - O Once a day
   - O More than once a week
   - O Once a week
   - O More than once per month
   - O Once a month
   - O Once in two months
   - O Other: ________________________
   - O Never (skip to item 15)
   - O No answer

13. **In the last 2 months, what were your main reasons for douching? (Fill in all that applies.)**
   - O Before seeing a doctor/nurse
   - O To avoid seeing a doctor/nurse
   - O To be fresh and clean
   - O To remove vaginal odor
   - O To remove menstrual blood
   - O To remove vaginal discharge
   - O To stop vaginal itching/irritation
   - O When I think I have an infection
   - O Before having sex
   - O After having sex
   - O Between sexual partners
   - O To please my partner
   - O To tighten my vagina before sex
   - O To prevent pregnancy
   - O No particular time
   - O I don't know why, I just do it
   - O Other: ________________________
   - O No answer

14. **In the last 2 months, what kinds of douches have you used? (Fill in all that applies.)**
   - O Store-bought solution, specify ________________________
   - O Water only
   - Homemade solution using:
     - O Baking soda
     - O Vinegar and water
     - O Clorox
     - O Lysol
     - O Pine-sol
     - O Other: ______________________
   - O Betadine
   - O Unknown
   - O No answer

15. **In the last 2 months, have you used any of the following feminine hygiene products? (Fill in all that applies.)**
   - O Feminine hygiene towelette
   - O Feminine hygiene spray
   - O Feminine hygiene powder
   - O Vaginal acid gel (RepHresh)
   - O Norform suppository
   - O Other: _____________________
   - O None (skip to item 16)
   - O No answer

   a. **How often did you use any of these feminine hygiene products?**
   - O More than once a day
   - O Once a day
   - O More than once a week
   - O Once a week
   - O More than once per month
   - O Once a month
   - O Once in two months
   - O Other: ______________________
   - O No answer

16. **In the last 2 months, did you use vaginal lubricants?** O No (skip to item 17) O Yes O No answer

   a. **What lubricant did you use? (Fill in all that applies.)**
   - O Saliva (spit)
   - O Vaseline
   - O Crisco/vegetable oil
   - O Glide
   - O KY jelly
   - O Other: ________________________
   - O No answer

Now, we will ask you some questions about your sexual activities because organisms from different objects or parts of your or your partner's body can get into your vagina during sex. Please answer these questions as honestly as you can.

Remember that you do not have to provide any answer you do not want to provide and any answer you do provide is confidential. Some of these questions may not apply to you, but we have to ask them to everyone.

17. **Have you ever used sex toys?** O No (skip to question 18) O Yes O No answer

   a. **In the last 2 months, how often have you used sex toys?**
   - O More than once a day
   - O Once a day
   - O More than once a week
   - O Once a week
   - O More than once per month
   - O Once a month
   - O Once in two months
   - O Other: ____________________
   - O Never
   - O No answer

18. **Have you ever had sex? By sex, we mean any sexual contact, including vaginal, oral and/or rectal contact.**
   O No (skip to question 28) O Yes O No answer

19. **How old were you when you first had sex?** _____ years old O No answer

20. **How many people have you had sex with in your life? (Give your best guess. If none, write 0 and skip to item 28.)**
   a. **Male:** _____ person or people O No answer
   b. **Female:** _____ person or people O No answer

21. **How many people have you had sex with in the last 2 months? (Give your best guess. If none, write 0 and skip to item 28.)**
   a. **Male:** _____ person or people O No answer
   b. **Female:** _____ person or people O No answer

22. **In the last 2 months, how often did you have vaginal intercourse?**
   O 1 time in 2 months
   O 1-3 times per month
   O 1-3 times per week
   O 4-6 times per week
   O 1-3 times per day
   O 4-6 times per day
   O 7-10 times per day
   O Never
   O No answer

23. **In the last 2 months, how often were you fingered?**
   O 1 time in 2 months
   O 1-3 times per month
   O 1-3 times per week
   O 4-6 times per week
   O 1-3 times per day
   O 4-6 times per day
   O 7-10 times per day
   O Never
   O No answer

24. **In the last 2 months, how often did you receive oral sex?**
   O 1 time in 2 months
   O 1-3 times per month
   O 1-3 times per week
   O 4-6 times per week
   O 1-3 times per day
   O 4-6 times per day
   O 7-10 times per day
   O Never
   O No answer

25. **In the last 2 months, how often did you receive anal sex?**
   O 1 time in 2 months
   O 1-3 times per month
   O 1-3 times per week
   O 4-6 times per week
   O 1-3 times per day
   O 4-6 times per day
   O 7-10 times per day
   O Never (skip to item 26)
   O No answer
   a. **Did you have vaginal sex right after having anal sex?** O No O Yes O No answer
   b. **Did your partner wear a condom when you had anal sex?** O No O Yes O No answer
   c. **Did your partner change condoms between anal and vaginal sex?** O No O Yes O No answer

26. **In the last 2 months, did you use feminine hygiene products before, during or immediately after sex?**
   O No (skip to item 27) O Yes O Not applicable O No answer
   a. **Which product(s) did you use? (Fill in all that applies.)**
      O Douche
      O Feminine hygiene towelette
      O Feminine hygiene spray
      O Feminine hygiene powder
      O Vaginal acid gel (RepHresh)
      O Norform suppository
      O Boric acid
      O Other: ________________________
      O No answer

27. **In the last 2 months, when you had sex, how often did you use a condom? (If your partner(s) is/are female, enter "Not applicable".)**
   O Always
   O Most of the time
   O About half the time
   O Seldom
   O Never
   O Not applicable
   O No answer

28. **Fill in all the methods of birth control you have used in the last 6 months. (See page 4 for the options.)**

   For each hormonal (*), semi-permanent and permanent method, provide a start, insertion or procedure date to indicate how long you have been using it. If you are no longer using it, provide an end or removal date.

   Cannot remember the date? If you remember the year, but not the month or the day, enter January 1, YYYY. If you remember the month and the year, but not the day, enter MO 1, YYYY.

   For each non-hormonal method, provide one of the following numbers to indicate how long you have been using it:

   1. **Less than 1 week**
   2. **1-2 weeks**
   3. **2-3 weeks**
   4. **3-4 weeks**
   5. **1-3 months**
   6. **3-6 months**
   7. **6-12 months**
   8. **1-3 years**
   9. **3-5 years**
   10. **5-10 years**
   11. **More than 10 years**
   12. **No answer**

- O *Pill, specify____________________ ➤ Start date: _______________ End date: _______________
- O *Patch, specify____________________ ➤ Start date: _______________ End date: _______________
- O *Ring, specify____________________ ➤ Start date: _______________ End date: _______________
- O *Injection, specify____________________ ➤ Start date: _______________ End date: _______________
- O *Implant, specify____________________ ➤ Insertion date: _______________ Removal date: _______________
- O (*)IUD, specify____________________ ➤ Insertion date: _______________ Removal date: _______________
- O Tubal ligation ➤ Procedure date: _______________
- O Partner's vasectomy ➤ Procedure date: _______________
- O Condoms for men, specify____________________ ➤ _____
- O Condoms for women, specify____________________ ➤ _____
- O Cervical cap ➤ _____
- O Diaphragm w/ jelly or foam, specify______________ ➤ _____
- O Diaphragm w/o jelly or foam, specify______________ ➤ _____
- O Spermicide, specify____________________ ➤ _____
- O Douche, specify____________________ ➤ _____
- O Morning-after pills, specify____________________ ➤ Last date: _______________
- O Basal body temperature rhythm ➤ _____
- O Ovulation calendar ➤ _____
- O Withdrawal ➤ _____
- O Abstinence ➤ _____
- O Other, specify____________________ ➤ _____ / Start date: _______________ End date: _______________
- O I am not currently using any birth control method.
- O No answer

Items 29 and 30 should only be answered if you are using a method of birth control with a * in front of it.

29. *Have you used this birth control as prescribed? (If "Yes", you did not miss any pills, you wore your patch or ring when you were supposed to, etc.) O No O Yes (skip to item 30) O No answer

a. **If "No", what percentage of pills did you miss, time did you not wear your patch or ring when you were supposed to, etc.?**
O 0-15% O 16-30% O 31-45% O 46-60% O 61-85% O 86-100%

b. **When did this "break" from your birth control start and end?** Start date: _______________ End date: _______________

30. *Have you experienced any irregular symptoms in the last 6 months? O No (skip to item 31) O Yes O No answer

If "Yes", please describe: ____

____

____

31. How old were you when you started using hormonal birth control?
_____ years old O I have never used hormonal birth control. O No answer

32. Have you ever been pregnant? O No (skip to end) O Yes O No answer

a. **How many live vaginal births have you had, *not* C-sections?**
O None O 2 O No answer
O 1 O More than 2

b. **How many live C-sections have you had?**
O None O 2 O No answer
O 1 O More than 2

c. **Have you ever miscarried?** O No O Yes O No answer

d. **Have you ever terminated one or more pregnancies?** O No O Yes O No answer

e. **When was your last live birth, C-section, miscarriage or termination?**

|____|____| / |____|____| / |____|____| (mm/dd/yy) O No answer

33. **Are you currently breastfeeding?** O No O Yes O No answer

Initials of person completing the form: _______________ Date: ____________________

Date: |____|____| / |____|____| / |____|____|	ENRL #: |__E__|____|____|____|

HCL Study – Enrollment Visit
Clinical Exam & Evaluation Form

For staff only: Site #: |____|____|____| Visit ID: |____|____|____|____| Staff ID: |____|____|

1. **HEIGHT ➤** |____|____| in (O Skipped)

2. **WEIGHT ➤** |____|____|____| lb (O Skipped)

3. **BP ➤** |____|____|____| / |____|____|____| mm Hg (O Skipped)

4. **SCLERA** (O Skipped)
   O White O Icteric O Other, specify____________________________

5. **ORAL EXAM** (O Skipped)
   O Normal O Decay
   O Sore(s) O Other, specify____________________________
   O Gingivitis

6. **NEUROLOGICAL EXAM** (O Skipped)
   O Focal O Non-focal O Other, specify____________________________

7. **CARDIOVASCULAR EXAM** (O Skipped)
   O Regular O Murmur O Other, specify____________________________

8. **PULMONARY EXAM** (O Skipped)
   O Normal O Abnormal, describe____________________________

9. **BREAST EXAM** (O Skipped)
   O Normal O Fibrocystic O Mass, describe____________________________

10. **ABDOMINAL EXAM** (O Skipped)
    O Normal O CVA
    O Tenderness O Mass, describe____________________________
    O Rebound O Other, specify____________________________

11. **EXTREMETIES** (O Skipped)
    O Normal O Edematous O Other, specify____________________________

12. **SKIN** (O Skipped)
    O Normal O Rash, describe____________________________
    O P&P rash O Molluscum
    O Folliculitis O Scabies
    O Intertrigo O Other, specify____________________________

13. **PUBIC HAIR** (O Skipped)
    O Normal O Crabs O Other, specify____________________________

14. **INGUINAL NODES** (O Skipped)
    O Normal O Enlarged & tender
    O Enlarged O Bilateral
    O Tender O Unilateral

15. VULVA/VAGINA (O Skipped)

- O Normal
- O Bartholin cyst
- O Edema
- O Erythema
- O Excoriations
- O Rash
- O Ulcer
- O Vesicle
- O Other, specify__________________________

16. Did the patient complain of any vaginal symptoms? (O Skipped)

- O No
- O Yes ➤
  - O Discharge
  - O Itching
  - O Foul odor
  - O Irritation
  - O Burning
  - O Other, specify________________

17. VAGINAL DISCHARGE (O Skipped)

- O No
- O Yes ➤

Location:

- O Vaginal walls +/- fornix
- O Fornix, posterior
- O Fornix, anterior
- O Fornix, both anterior and posterior

Consistency: O Homogenous (smooth) O Inhomogenous (clumpy)

Color:

- O Clear
- O Yellow/green
- O Other, specify________________
- O White/cream/gray
- O Bloody

Character: O Thin, flows O Thick, does not flow O Other, specify____________________________

Quantity: O Scant (little on speculum) O Moderate (speculum <½ full) O Large (speculum >½ full)

18. CERVIX (O Skipped)

- O Normal
- O Cervical discharge ➤ O Clear O Cloudy white O Yellow/green O Bloody O No answer
- O Ulcer
- O Vesicle
- O Friability ➤ O Mild (blood spots) O Moderate (soaked with blood)
- O Fundal tenderness
- O Ectopic zone ➤ O <25% O 26-50 O 51-75 O >75

19. BIMANUAL EXAM (O Skipped)

Adnexal:

Tenderness ➤	O Right	O Left	O Bilateral	O N/A
Fullness ➤	O Right	O Left	O Bilateral	O N/A
Mass ➤	O Right	O Left	O Bilateral	O N/A

Fundal:

Cervical tenderness ➤	O No	O Yes
Uterine enlargement ➤	O No	O Yes
Mass/fibroids ➤	O No	O Yes

20. RECTUM (O Skipped)

- O Normal
- O Discharge
- O Fissure
- O Hemorrhoids
- O Ulcer
- O Warts
- O Other, specify__________________

21. AMSEL CRITERIA

Vaginal pH ➤	O 4.0	O 4.7	O 5.3	O 5.8	O 6.5	(O Skipped)
	O 4.4	O 5.0	O 5.5	O 6.1	O 7.0	
WHIFF test ➤	O Negative	O Positive				(O Skipped)
Homogenous discharge ➤	O No	O Yes				(O Skipped)
Clue cells ➤	O No	O Yes				(O Skipped)

22. DIAGNOSIS **(O Skipped)**

O BV, asymptomatic
O BV symptomatic
O Gonorrhea
O Herpes
O MPC
O PID
O Syphilis
O Trichomonas
O Genital warts
O Yeast
O Other, specify__________________
O None

22. What medication(s) was prescribed? (O Skipped)

O Metronidazole, 500 mg bid x 7 days
O Metronidazole, 2 g orally, at once
O Metronidazole gel 0.75%, one full applicator (5 g) intravaginally, qd x 5 days
O Clindamycin cream 2%, one full applicator (5 g) intravaginally, at bedtime x 7 days
O Anti-fungal topical cream, specify_____________________________
O Fluconazole, 150 mg orally
O Other, specify_____________________________
O None

23. SPECIMENS (Fill in all collected.)

O 30 ml blood
O 5 ml saliva
O Urine
O GenProbe vaginal swab
O Copan vaginal swab **1**
O Copan vaginal swab **2**
O Starplex vaginal swab
O dacron vaginal swab
O dacron ectocervical swab
O dacron endocervical swab
O endocervical brush
O Copan rectal swab
O vaginal smear
O Pap smear
O None

24. REAL-TIME PREGNANCY TESTING

O No
O Yes ➤ Performed by... O the OB/GYN clinic O the study team

➤ Result is… O Negative O Positive

25. REAL-TIME STD TESTING

O No
O Yes ➤ Performed by... O the OB/GYN clinic O the study team

➤ Testing for…

O Gonorrhea
O Chlamydia
O Trichomonas
O Mycoplasma
O Syphilis
O Herpes
O Genital warts (HPV)
O Other, specify_____________

➤ Result is… O Negative O Positive, specify_____________________

26. REAL-TIME HIV TESTING

O No
O Yes ➤ Performed by... O the OB/GYN clinic O the study team

➤ Result is… O Negative O Positive

Initials of person completing the form: ________________ Date: _____________________
